# Enhanced HDR-mediated correction of heterozygous *COL7A1* mutations for recessive dystrophic epidermolysis bullosa

**DOI:** 10.1016/j.omtn.2025.102472

**Published:** 2025-02-01

**Authors:** John M.T. Hunt, Alex du Rand, Daniel Verdon, Leah Clemance, Evert Loef, Chloe Malhi, Ben Buttle, David J.H.F. Knapp, Yale S. Michaels, Jonathan Garlick, P. Rod Dunbar, Diana Purvis, Vaughan Feisst, Hilary Sheppard

**Affiliations:** 1 School of Biological Sciences, The University of Auckland, Auckland 1010, New Zealand; 2 Maurice Wilkins Center, New Zealand; 3 Institut de recherche en immunologie et en cancérologie (IRIC) and Département de Pathologie et Biologie Cellulaire, Université de Montréal, Montréal, QC H3T 1J4, Canada; 4 Paul Albrechtsen Research Institute CancerCare Manitoba, Winnipeg, MB R2H 2A6, Canada; 5 Department of Biochemistry and Medical Genetics, Rady Faculty of Health Sciences, University of Manitoba, Winnipeg, MB R3E 0J9, Canada; 6 School of Dental Medicine, Tufts University, Boston, MA 02111, USA; 7 Te Whatu Ora Health New Zealand, Te Toka Tumai, Auckland 1023, New Zealand

**Keywords:** MT: RNA/DNA Editing, CRISPR-Cas9, gene therapy, epidermolysis bullosa, skin engineering, homology-directed repair, dual-nickase, Oxford Nanopore Technology sequencing

## Abstract

Gene editing facilitated by homology-directed repair (HDR) holds great potential for treating monogenetic disorders such as recessive dystrophic epidermolysis bullosa (RDEB). However, low efficiency and variability between loci must be overcome for its widespread adoption into personalized therapies. To address these challenges, we developed a highly efficient and versatile gene editing strategy for RDEB that incorporates the small molecule inhibitor M3814 to enhance HDR. We focused on three RDEB causative *COL7A1* mutations not previously targeted by existing gene therapies. Editing was achieved using Cas9-nuclease ribonucleoproteins with short single-stranded DNA donor templates, and outcomes were assessed with an Oxford Nanopore Technology sequencing analysis pipeline. We demonstrate precise genomic HDR rates of up to 75% of alleles in primary RDEB keratinocytes and 32% in fibroblasts. This approach restored collagen VII expression in up to 80% of keratinocytes within a bulk-edited population and resulted in correct collagen VII deposition in a 3D skin model. Additionally, at one locus we show that a dual Cas9-nickase strategy is less effective than Cas9-nuclease and prone to large on-target deletions. Our results demonstrate a significant advancement in the efficiency and consistency of HDR editing, potentially paving the way for more effective personalized gene therapies.

## Graphical abstract

**Figure:**
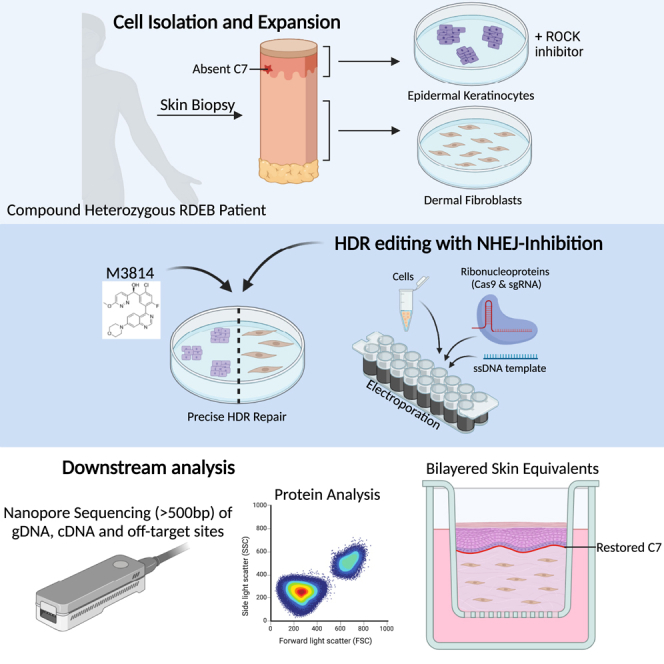

## Teaser

Hunt and colleagues present an enhanced gene editing strategy for treating RDEB. Targeting three unique mutations they achieve up to 75% gene correction in bulk-edited cells and demonstrate protein restoration in lab-grown skin models. This advances potential therapies for this inherited skin disorder.

## Introduction

Epidermolysis bullosa (EB) is a group of inherited skin disorders, characterized by fragility of mucosal and cutaneous membranes. Recessive dystrophic EB (RDEB) represents one of the most clinically severe EB subtypes and results from mutations within the *COL7A1* gene encoding for the c**ollagen alpha-1(VII) chain** (C7) protein.^1^ C7 is the main component of anchoring fibrils, which are deposited at the basement membrane zone (BMZ) and are essential for adherence between the epidermal and dermal skin layers.^1^ In RDEB, the bi-allelic loss of functional C7 results in severe, poorly healing blisters that can be accompanied by other complications such as cutaneous squamous cell carcinomas, the leading driver of morbidity and mortality in this cohort.^2^

Currently, no permanent cure exists for RDEB. While various therapeutic strategies are being investigated,^3^ the correction of causative mutations would be ideal for a long-term therapy. In this regard, the CRISPR-Cas genome editing system stands out with its potential for inexpensive and robust editing. It relies on a site-specific Cas9 endonuclease which can be targeted to cleave double-stranded DNA at almost any user-defined locus. Target specificity is provided by a programmable guide RNA (gRNA) sequence that is homologous to the target genomic region; DNA cleavage only occurs if the target locus is adjacent to a Cas9-variant-dependent protospacer adjacent motif (PAM).^4^ In human cells double-stranded DNA breaks (DSBs) are resolved by three main mechanisms: the error-prone non-homologous end-joining (NHEJ) and microhomology-mediated repair (MMEJ) pathways, or the high-fidelity homology-directed repair (HDR) pathway.^5^

Gene editing with HDR is a preferred strategy compared with NHEJ or MMEJ as it has the potential for precise correction of most mutations.^5^ Over recent years, methods have evolved to yield increasingly higher rates of HDR in *COL7A1.*^6^^,^^7^^,^^8^^,^^9^^,^^10^^,^^11^ Recently, Bonafont et al. (2021)^6^ achieved up to 50% HDR at the genomic level when targeting a homozygous c.6527insC mutation, using Cas9 delivered as a ribonucleoprotein complex (RNP) with an adeno-associated virus 6 donor template. While efficient, the use of viral vectors during editing has been associated with high rates of random or on- and off-target vector integration into the genome.^12^ Co-delivery of single-stranded DNA (ssDNA) donor templates with RNPs offers a simpler solution while also reducing the risk of these integration events.^12^ Using these methods, Berthault et al. (2023)^7^ were able to achieve up to 58% HDR of genomic DNA when targeting a homozygous c.6508C>T mutation in primary RDEB keratinocytes. However, only 4 of 37 analyzed PCR amplicons had perfect HDR-edited alleles without also containing indels. These contaminating indels limit the use of this method to intronic gRNA sites. An alternative HDR-approach relies on the use of dual-nickase to create a staggered dsDNA break, which has the added benefit of reduced off target effects compared with Cas9 nuclease.^13^ Kocher et al. (2019, 2021)^8^^,^^9^ described a dual nickase approach to repair a homozygous c.425A>G mutation. Here, they reported rates of up to 21% precise HDR at the gDNA level in primary keratinocytes and up to 10% HDR in primary fibroblasts. Although these results are encouraging, they may fall short of the repair rates required for long-term clinical efficacy, which are estimated to be 20%–30%.^9^^,^^10^ Recently, we described a dual nickase approach targeting *LAMB3* for junctional EB and achieved up to 54% HDR frequency.^14^ This suggests that nickases may be a clinically viable option for some loci.

In this study, we describe a highly efficient, precise, and versatile method for enhanced Cas9-nuclease-mediated HDR editing using the NHEJ inhibitor M3814. We demonstrate clinically useful rates of gene repair of one unique and two described but previously untargeted heterozygote *COL7A1* mutations present in three people with RDEB from New Zealand. We achieved up to 75% precise, HDR-mediated repair at the gDNA level in early passage primary RDEB keratinocytes and 32% in RDEB fibroblasts. Gene-corrected RDEB cells showed restored C7 expression *in vitro* and C7 deposition to the BMZ in engineered 3D skin equivalents (SEs). Additionally, a dual-nickase strategy executed in RDEB keratinocytes in the presence of M3814 achieved up to 44.5% HDR-mediated repair at the genomic level. However, this approach was hampered by frequent large on-target deletions. Our findings expand the field of gene therapy research for RDEB and demonstrate a promising advancement in targeted *COL7A1* gene repair mediated by HDR.

## Results

### Design of CRISPR single-gRNAs and HDR templates for the correction of three heterozygous RDEB mutations

**Figure 1:**
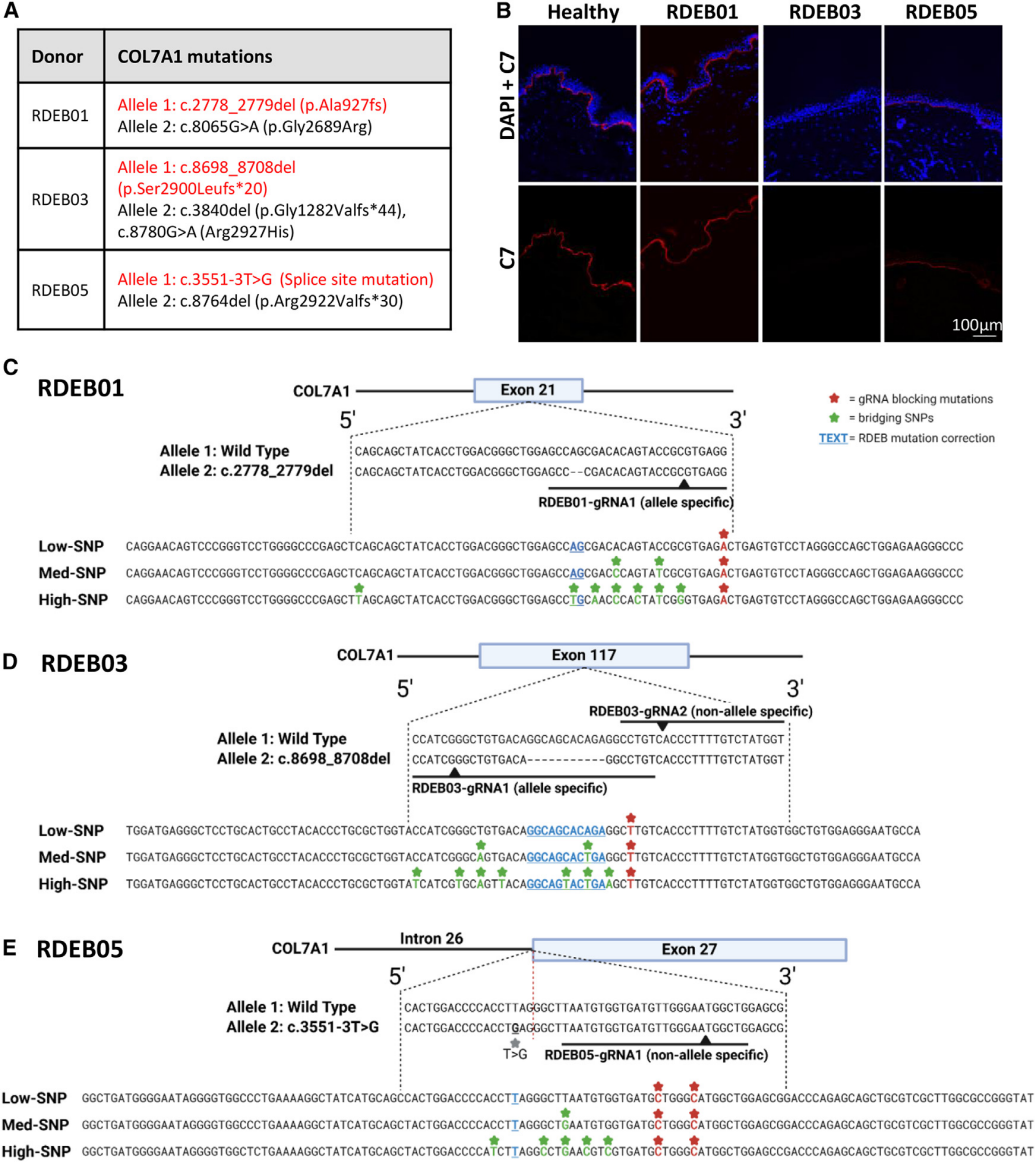
HDR-mediated editing strategy for three *COL7A1* mutations (A) Annotation of *COL7A1* mutations (transcript GenBank: NM_000094.3) from three RDEB donors, with targeted alleles highlighted in red. (B) Immunohistochemistry analysis of skin punch biopsy from one healthy and three RDEB donors, showing DAPI-stained nuclei in blue and C7 in red. Scale bar (bottom right), 100 μM. (C–E) HDR-based editing reagent design for three heterozygous *COL7A1* mutations. The approximate mutation locations within *COL7A1* exons are shown. Both WT and MUT alleles are depicted for each locus. For RDEB01 and RDEB03 (C and D), deletion mutations are denoted with dashed lines. For RDEB05 (E), the substitution mutation is marked in bold and indicated by a gray asterisk. Black lines represent gRNA binding sites, with cut sites shown as black arrow heads and allele specificity is indicated. HDR templates are displayed below, showing mutation correction as underlined blue bases, gRNA blocking mutations as red bases (with asterisks), and bridging SNPs as green bases (with asterisks).

Here we present gene editing results from experiments performed on cells derived from three New Zealand donors with RDEB, referred to hereafter as RDEB01, RDEB03, and RDEB05. Each donor had at least two confirmed or predicted pathogenic compound heterozygous mutations in the *COL7A1* gene (Figure 1A). Immunohistochemistry analysis of donor skin punch biopsies revealed normal levels of C7 expression in RDEB01, reduced but detectable C7 in RDEB05, and undetectable C7 in RDEB03, as compared with normal human skin (Figure 1B). Donor RDEB01 is diagnosed with RDEB inversa, a form of RDEB characterized by blistering localized to body areas of higher temperature, such as the axillae, groin, oral, and esophageal regions.^15^ In this donor, the missense mutation c.8065G>A causes a glycine substitution within the triple-helical domain of C7, which is known to affect the thermostability of the C7 complex.^15^ Therefore, while the C7 protein is abundant in the RDEB01 skin biopsy, it likely has reduced thermostability, leading the RDEB inversa pathology.

We focused our attention on mutations located in exons present in the non-collagenous domains (NC-1 and NC-2) domains of *COL7A1*, hypothesizing that these are less likely to be amenable to an exon skipping strategy.^16^ In donors RDEB01 and RDEB03, the two described mutations c.2278_2279del and c.8698_8708del^16^ result in a frameshift and the formation of a premature stop codon in *COL7A1* exon 21 and exon 117, respectively. In donor RDEB05, c.3551-3T>G is a splice-site mutation in the splice acceptor site of intron 26. An analysis of *COL7A1* mRNA transcripts from this donor identified that this mutation results in a previously unreported sequential skipping of exons 26–37 (Figure S1).^17^

For each of these three mutations, single-gRNAs (sgRNAs) and short single-stranded oligonucleotide templates (HDR templates) were designed for use with spCas9 nuclease (Cas9) (Figures 1C–1E). sgRNAs were selected based on the proximity of the predicted cut site to the patient mutation and were screened based on Cas9 cutting efficiency in wild-type (WT) and RDEB derived keratinocytes (data not shown). Of the four selected sgRNAs, two were specific to the mutant (MUT) allele (RDEB01 gRNA1 and RDEB03 gRNA1) and two were non-specific targeting both alleles (RDEB03 gRNA2 and RDEB05 gRNA2). Allele specificity was confirmed by recording 0% editing (indels) in homozygous WT cells (data not shown), and by consistently recording 0% editing of the WT allele in RDEB patient cells (see Figure S2).

HDR templates were designed to revert the causative mutations to WT sequence as well as incorporate a blocking mutation to the gRNA PAM site to prevent recutting after HDR (Figures 1C–1E). We recently demonstrated enhanced editing rates in primary human hematopoietic stem cells using HDR templates containing multiple silent mutations.^18^ Therefore HDR templates were designed with increasing numbers of SNPs to assess whether a similar effect could be achieved in cells derived from the skin, as follows: low SNPs (1–2 SNPs), medium SNPs (3 SNPs), and high SNPs (8–9 SNPs), hereafter referred to as low-SNP, med-SNP, and high-SNP, respectively (Figures 1C–1E). For RDEB05-gRNA2 including silent mutations within the PAM-site itself was not possible, so instead two SNPs were placed within the gRNA sequence in proximity to the PAM site. The gene editing reagents were delivered concomitantly as RNP complexes and ssDNA via electroporation.

### Validation of an on-target editing analysis pipeline with Oxford Nanopore Technologies sequencing

Conducting a robust on-target analysis of bulk-edited DNA poses several challenges. Sanger sequencing of bulk or cloned DNA can lack sufficient analytical accuracy, especially at heterozygous loci, necessitating more expensive next-generation sequencing methods. Additionally, standard short-read Illumina sequencing is constrained to sequences around 150–300 base pairs, potentially missing the full editing landscape at a target site. To address these issues, we implemented an Oxford Nanopore Technology sequencing (ONT-seq) pipeline capable of deep sequencing a larger PCR amplicon covering our target sites, and used the CRISPResso2 pipeline, which was originally designed for Illumina sequencing, for our purposes.^19^

CRISPResso2 provides comprehensive reports on unedited alleles, indels, and HDR events, including detailed percentage breakdowns, alignment data, and indel distribution histograms. It categorizes HDR into two types: HDR, which includes reads closest to a perfect HDR sequence, and HDR-edited, which includes HDR events with contaminating indels likely derived from imperfect-HDR or sequencing errors. Additionally, it identifies ambiguous reads that align equally well with multiple defined alleles. In our analysis, we report HDR percentages based solely on the HDR category. We exclude HDR-edited and ambiguous reads, noting that these categories may harbor editing events that restore protein expression. Thus, our reported rates may under-represent the total of clinically useful editing rates.

**Figure 2:**
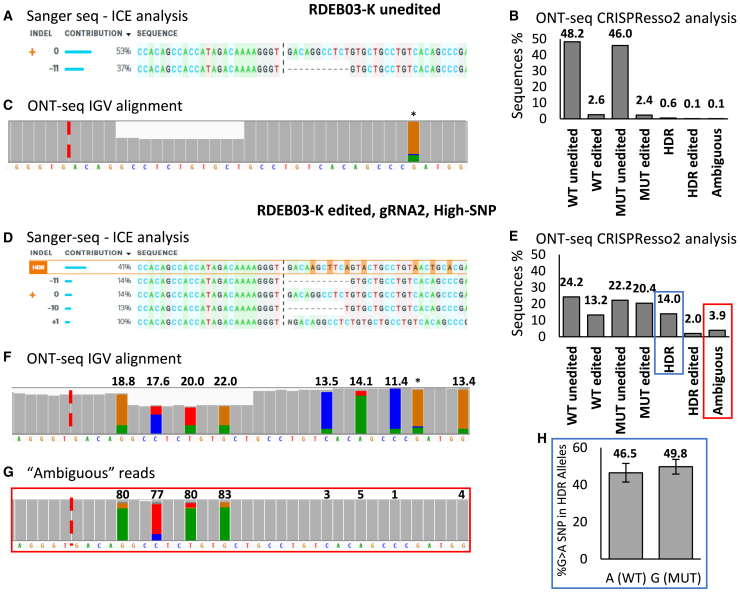
ONT-seq enables robust analysis of on-target editing events (A–C) Analysis of bulk DNA from unedited RDEB03 keratinocytes. (A) ICE analysis of Sanger sequencing data. Allele frequencies are displayed, where 0 and −11 represent the WT and MUT (c.8698_8708del) alleles, respectively. The gRNA cut site is marked by a vertical dashed line. (B) Allele frequencies reported as percentages from CRISPResso2 analysis of ONT-seq data. Categories include: WT unedited, WT edited, MUT unedited, MUT edited, HDR, HDR edited, and ambiguous. WT refers to the heterozygous allele, while MUT corresponds to the allele containing the c.8698_8708del mutation. Ambiguous reads align equally to multiple alleles and are given a distinct category. (C) Raw ONT-seq reads are aligned to the reference genome and visualized using the IGV. The histogram summarizes the depth of alignment for each base, with deletions represented by a proportional decrease in gray bar height and substitutions that exceed a 10% frequency threshold highlighted by a change in color. The Cas9 cut site is denoted by a dashed red line, and a frequent nanopore sequencing error is marked with an asterisk. (D–G) Analysis of bulk DNA from RDEB03 keratinocytes edited with gRNA2 (non-allele-specific) and the Full-SNP HDR template. (D) As described in (A), with HDR events highlighted in orange. (E) As described in (B). (F) As described in (C), with HDR-induced silent mutations highlighted, and their frequencies indicated above. (G) Reads classified as ambiguous by CRISPResso2 in panel E (boxed in red) were extracted, aligned to the reference genome, and visualized in IGV. (H) Reads that were classified as HDR by CRISPResso2 in (E) (boxed in blue) were extracted and the allelic frequency of a nearby heterozygous SNP (c.8780G>A) present in *cis* with the WT allele were calculated (*n* = 4). Adenine (A) is on the WT allele, and guanine (G) on the MUT allele.

To validate the ONT-seq and CRISPResso2 pipeline for HDR analysis at a heterozygous locus, RDEB03 derived keratinocytes (RDEB03-Ks) were edited with the RDEB03-high-SNP HDR template and guide RDEB03-gRNA2, which targets both the WT and MUT alleles. Sanger sequencing and ONT-seq were then performed on the same input amplicon DNA flanking the *COL7A1* exon 117 deletion (c.8698_8708del). Sanger data were analyzed using Inference of CRISPR Edits (ICE) software (from Synthego) and the ONT-seq data were analyzed with CRISPResso2 (Figure 2). Analysis of an unedited control amplicon returned an expected approximately 50% WT reads (53% and 48.2% comparing ICE with CRISPResso2 data, respectively), but ICE under-reported the presence of the MUT allele (37% compared with 46%, respectively) and misaligned the gRNA cut site (Figures 2A and 2B). An advantage of the ONT-seq data/CRISPResso2 analysis is that it provides data for the percentage of reads in the following categories: WT unedited, WT edited, MUT unedited, MUT edited, HDR, HDR edited, and ambiguous (Figure 2B). We note that the unedited RDEB03 control sequences reported a false indel rate of approximately 2.5% (2.6% and 2.4% for WT and MUT alleles) (Figure 2B), which was consistent with the expected error rate at that time from ONT-seq of approximately 1% per nucleotide, with indels identified using a two-nucleotide window around the cut site. The long read data can also be aligned to the reference genome using Integrative Genomics Viewer (IGV), providing a clear visual of read coverage for each base sequenced, which clearly shows the 11-bp deletion present in the unedited sample (Figure 2C).

When these tools were used to analyze edited DNA, the estimated HDR rates differed substantially, with ICE estimating 41% and CRISPResso2 reporting 14% (Figures 2D and 2E). Alignment of the ONT-seq data revealed unequal insertion of the eight HDR SNPS, ranging from 11.4% to 22.0% insertion rates, where the four SNPs proximal to the gRNA2 cut site were introduced more frequently than the four distal SNPs (Figure 2F). Notably, CRISPResso2 classified 3.9% of the edited reads as ambiguous (Figure 2E), which represented these partial HDR events (Figure 2G). These reads aligned equally with WT and HDR allele sequences, resulting in their ambiguous classification.

Patient RDEB03 carries a heterozygous SNP in *COL7A1* exon 117 (c.8780G>A), which is present in the sequenced amplicons. The SNP is located in *trans* to the targeted 11-bp deletion (c.8698_8708del) and can be used as a marker to trace the parental allele origin of HDR events when using non-allele-specific gRNAs. To confirm that HDR events were equally distributed between the MUT and WT alleles, and that the reported HDR rates accurately reflect the expected rate of cellular correction, reads classified as HDR by CRISPResso2 were analyzed for the presence of the c.8780G>An SNP. For samples edited with a non-allele-specific guide (gRNA2) and various HDR templates, the G>A SNP was observed at an average frequency of 46.5% and 49.8% for A (WT) and G (MUT), respectively, with no significant difference observed (Figure 2H). This confirmed an equal distribution of HDR events across both parental alleles, and this distribution was then assumed in donors where such an analysis was not possible. Further certainty was provided later when we observed a strong linear correlation between the ONT-seq/CRISPResso2 HDR estimates and the percentage of C7-positive cells, further validating this analysis pipeline (see Figure 4D).

These results demonstrate that the ONT-seq/CRISPResso2 analysis pipeline outperforms Sanger-seq/ICE analysis when examining heterozygous mutations. Furthermore, CRISPResso2 effectively processed ONT-seq data, enabling a robust analysis of gene editing. Herein, HDR efficiency is presented as a percentage of the reported MUT allele so that the displayed efficiencies represent the predicted rate of cellular correction. Furthermore, imperfect-HDR (with indels) or ambiguous reads which may result in functional editing are not included when reporting HDR rates, thus the reported rate may slightly underrepresent the actual rate of repair.

### Small molecule inhibition of DNA-activated protein kinase significantly improves the efficiency of targeted gene editing in primary RDEB cells

**Figure 3:**
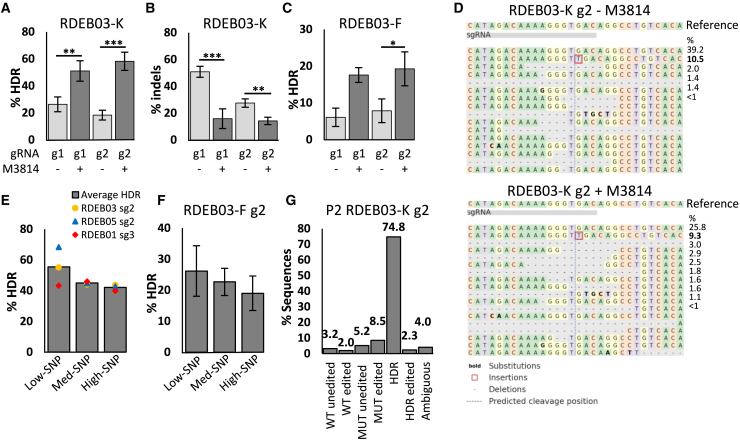
High rates of on-target HDR-mediated gene editing is achieved in skin cells derived from donors with RDEB Keratinocytes and fibroblasts, derived from three donors with RDEB, were gene edited with Cas9-nuclease and HDR templates as indicated. HDR rates were measured by ONT-seq with CRISPResso2 analysis. (A) Calculated HDR rates for the MUT allele and (B) total indel rates are shown from bulk RDEB03-Ks edited with one of two sgRNAs as shown, the RDEB03 high-SNP template, with or without M3814. Data are presented as mean ± SD (*n* = 3, ∗*p* < 0.05, ∗∗*p* < 0.01, ∗∗∗*p* ≤ 0.001). (C) HDR rates in RDEB03 fibroblasts (RDEB03-Fs) edited with one of two sgRNAs and the RDEB03 high-SNP template and with or without M3814 (*n* = 2 for gRNA1, *n* = 3 for gRNA2). (D) Screen capture of CRISPResso2 allele alignment showing indel distribution in RDEB03-Ks edited with RDEB03 gRNA2 and the high-SNP template, with or without M3814. This alignment only includes reads which align closest to the c8698_8708del allele (MUT-unedited and MUT-edited) (see Figure 2). The reference allele is at the top, with indels below in decreasing frequency (displayed to <1%). The gRNA cut site is marked by a vertical dashed black line. Substitutions are in bold, insertions are outlined in red, and deletions are marked as dots. (E) Calculated HDR rates for the MUT allele in bulk keratinocytes derived from three RDEB donors comparing three HDR templates for each donor. The data are presented as an average percentage rate for each HDR-template, with individual donor rates indicated (RDEB03 *n* = 2, RDEB01 and RDEB05 *n* = 1). (F) HDR rates in RDEB03 fibroblasts edited with three different HDR templates (*n* = 2). (G) CRISPResso2 analysis output from low passage (P2) RDEB03 keratinocytes edited with gRNA2 and low-SNP HDR template (*n* = 1).

Previous reports suggest that potent inhibition of DNA-activated protein kinase (DNA-PK) with nedisertib (M3814) enhanced HDR rates in immortalized cell lines^18^^,^^20^ and human hematopoietic stem cells.^18^ To assess its effect in primary human skin cells, and following titration experiments, we repeated gene editing experiments in RDEB03-Ks and RDEB03-Fs in the presence of 1 μM M3814 for 72 h. A marked increase in HDR rates was observed in the presence of M3814 (Figure 3), with no noted difference in cell viability (data not shown). Exposing RDEB03-Ks to M3184 during editing with a MUT allele-specific guide (gRNA1) increased HDR rates from an average of 26.3%–51.1% (*p* = 0.02) (Figure 3A), while indels decreased from an average of 50.9%–15.9% (*p* = 0.004) (Figure 3B). With a non-allele-specific guide (gRNA2), HDR rates increased from an average of 15.06%–47.71% (*p* < 0.001) (Figure 3A) and indels decreased from 27.5% to 14.2% (*p* = 0.011) (Figure 3B). Similarly, when editing RDEB03-Fs with gRNA1 M3814 exposure increased HDR rates from 6.09% to 17.59% (*p* = 0.07), and with gRNA2 from 7.86% to 19.27% (*p* = 0.047) (Figure 3C). Interestingly, despite the increase in HDR rates in RDEB03-Fs, indel formation was not significantly reduced in the presence of M3184 (Figures S2 and S3). In all but one case (RDEB03-Ks treated with gRNA2), M3814 selectively enhanced HDR rates, without affecting the rates of total editing (HDR + indels) (Figure S2). This suggests that the inhibition of DNA-PK pushes the balance of DNA repair toward HDR-mediated repair, rather than increasing the amount of total editing.

The HDR rates reported by CRISPresso2 represent pure HDR with no contaminating indels. Therefore, to examine if the presence of M3814 affects the composition of indels we assessed the MUT-allele data subset (Figures 3D and S3). Here the most abundant indel, either in the presence or absence of M3814, was a single insertion of thymine (Figure 3D). However, in cells edited in the presence of M3814, there was a decrease in unedited alleles (perfect end-joining) and small increase in the proportion of deletions sized 10–50 base pairs (Figure S3). No notable increase in deletions larger than 50 base pairs was observed (Figure S3). These observations were consistent between cell types and on both alleles (WT and MUT).

To assess the impact of HDR template design, we next edited keratinocytes from the three RDEB donors with HDR templates incorporating different numbers of silent SNPs (as per the design illustrated in Figures 1C–1E). Editing in the presence of M3814 resulted in average HDR rates of 40%–68% across all template designs (Figures 3E and S4). No statistical difference was observed between the template designs for each RDEB donor, nor when data from all donors were analyzed using a two-way ANOVA test. However, for RDEB03 and RDEB05, the low-SNP template resulted in more than 10% higher HDR rates compared with the med-SNP and high-SNP templates. In RDEB03-Fs, average HDR rates between 20% and 26% were achieved across the different template designs (Figure 3F), but no significant differences were observed. Further replicates to potentially achieve significant results were precluded due to the limited availability of these primary cells.

The data presented in Figures 3A–3F were generated using late passage cells (passages 5–8). Next, we applied our optimized editing strategy to lower passage RDEB03-Ks (passage 2), which are likely to contain higher levels of stem cell-like progenitor cells and fewer clonally differentiated cells.^21^ In these cells, we observed 74.8% HDR when editing with the low-SNP HDR template in the presence of M3814 (Figure 3G). Again, replicate experiments were not conducted due to the limited availability of these low passage primary cells. No passage number effect was noted when editing was performed in fibroblast cells which likely reflects their more constant cell cycle over time in *in vitro* culture.^22^

Finally, to screen for potential off-target editing activity of the Cas9-nuclease, we performed ONT-seq/CRISPResso2 analysis of the top four predicted off-target sites for RDEB03 gRNA2 (Figure S5). Analysis of edited RDEB03-Ks with or without M3814 revealed no notable off-target editing at these sites compared with the unedited control sequences.

### HDR-mediated gene repair results in high levels of C7 expression in bulk-edited RDEB keratinocytes

**Figure 4:**
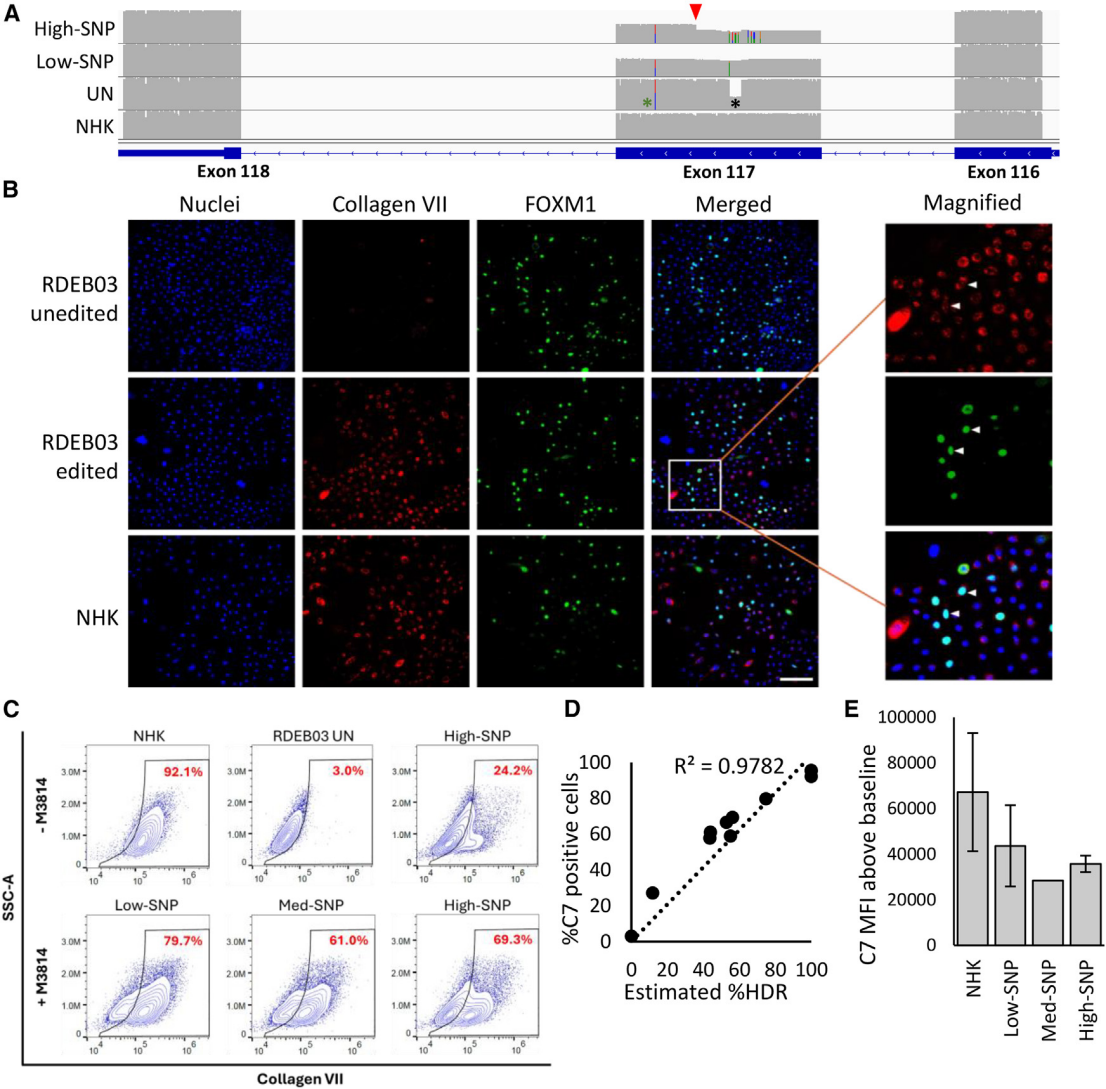
*COL7A1* transcription and C7 protein analysis in HDR-edited RDEB cells (A) *COL7A1* transcript analysis: ONT-seq data of *COL7A1* transcripts aligned to the reference genome, displayed as coverage tracks in IGV. Rows from top to bottom: Transcripts from RDEB03-Ks edited with either high-SNP or low-SNP HDR templates, unedited RDEB03-Ks (UN), and normal human keratinocytes (NHKs). The target 11bp deletion (c.8698_8708del) is indicated with a black asterisk, the green asterisk indicates the non-target mutation (c.8780G>A) on the opposite allele. An aberrant splice site is marked by a red arrow. (B) Immunocytochemistry analysis of C7 (in red) in unedited and edited RDEB03-Ks and NHKs as labeled. RDEB03-Ks were edited with low-SNP template with M3814. Nuclei are stained with DAPI (blue). Nuclear FOXM1 staining (green) marks potential progenitor cells. Merged images are shown on the right-hand panel, where cyan represents merged DAPI (blue) and FOXM1 (green) nuclear stains. Scale bar (bottom right), 100 μM. On the far right, images are enlarged with white arrows indicating cells co-stained for FOXM1 and C7. (C) Flow cytometry analysis of C7 expression in HDR-edited RDEB03-Ks. Plots show C7 expression on the x axis and side scatter (SSC-A) on the y axis. The percentage of C7-positive cells is indicated in red text. (Top row) NHKs, unedited RDEB03-Ks (UN), and high-SNP edited RDEB03-Ks without M3814. (Bottom row) RDEB03-Ks edited with low-SNP, med-SNP, and high-SNP HDR templates with M3814. (D) Linear correlation (R^2^ = 0.9782) between HDR efficiency as assessed by ONT-seq/CRISPResso2 (x axis) and the percentage of C7-positive cells as assessed by flow cytometry (y axis). The y intercept set to 0. (E) The average mean fluorescence intensity (MFI) of the intracellular C7 signal above the baseline of unedited RDEB03 cells in NHKs (*n* = 3), RDEB03-Ks edited with the Low-SNP (*n* = 3), Med-SNP (*n* = 1), and High-SNP templates (*n* = 3).

We hypothesized that the introduction of multiple silent SNPs by HDR would not impact the translation of mRNA into protein; however, a potential concern is that SNPs might activate cryptic splice sites, leading to aberrant mRNA splicing. To verify correct splicing after HDR editing, we analyzed *COL7A1* transcripts from edited RDEB03 cells by ONT-seq cDNA samples generated from mRNA. We identified a native splice variant in transcripts from NHKs and unedited RDEB03-Ks, characterized by complete skipping of exon 117 (Figure 4A, showing fewer reads through exon 117 compared with flanking exons). This splice variant comprised around 5.6–8.7% of reads in unedited cells and increased to 30–50% in all editing conditions, irrespective of the indel composition, HDR template used or the presence of the M3814 inhibitor (Figures 4A and S6). Furthermore, analysis of cDNA also revealed aberrant splicing in up to 15.8% transcripts from cells edited with the high-SNP HDR template, whereas no such aberrations were observed in cells edited with the low-SNP HDR template (Figure 4A). Further inspection indicated that this aberrant splicing involved the adoption of a new endogenous splice acceptor (AG) site once HDR SNPs were introduced (Figure S6). Comparable findings were also observed in RDEB03-Fs (Figure S6).

In low-passage RDEB03-Ks edited with the low-SNP template and treated with M3814, correctly spliced and accurately edited transcripts accounted for 41.17% of the total transcripts (Figure 4A). Of the transcripts containing exon 117, the proportion with precise HDR editing was 74.2%, closely aligning with the ONT-seq HDR estimate of 74.8% (Figure 3G).

To assess the impact of editing on the expression of the C7 protein, we analyzed bulk-edited keratinocyte populations using immunocytochemistry, which revealed a high proportion of C7-expressing cells (Figures 4B and S7). In an attempt to verify that we had corrected epidermal stem cells, we co-stained RDEB03-Ks with anti-C7 and anti-FOXM1 antibodies, as FOXM1 is a putative keratinocyte stem cell marker.^23^ No differences in FOXM1 expression were observed by a visual comparison of NHKs to edited or unedited RDEB03-Ks (Figure 4B). In the edited samples, co-staining of C7 and FOXM1 was apparent, indicating that gene correction of potential progenitor cells had occurred (Figure 4B, magnified panels). Similar results were obtained with edited RDEB05-Ks (Figure S8).

To further evaluate C7 expression in edited RDEB cells, we performed a quantitative analysis using flow cytometry (see Figure S9 for gating strategy). Normal human keratinocytes contained a high proportion of C7-positive cells, ranging from 89% to 92%, in contrast with only 3% in unedited RDEB03-K s (Figures 4C and S10). After HDR repair, all edited RDEB03-Ks contained a distinct C7-positive population (Figures 4C and S10). Specifically, low-passage RDEB03-Ks edited with the low-SNP template exhibited a C7-positive population of 79.7% which is similar to the estimated HDR rate of 74.8% obtained through ONT-seq (Figure 3G). Indeed, a strong linear correlation (R^2^ = 0.978) was observed when comparing the estimated percentage of gene corrected cells (gDNA analysis) with the observed percentage of C7-positive cells (flow cytometry analysis) (Figure 4D). An intracellular staining protocol was used in this analysis, so the mean fluorescence intensity (MFI) data reflect the average expression level of intracellular non-secreted C7 (Figure 4E). No significant differences in fluorescence intensity were observed when comparing RDEB03-Ks corrected with the HDR template variants. However, the edited RDEB03-Ks exhibited reduced fluorescence intensity compared with WT keratinocytes (on average approximately 50%, MFI 35,950 cf. 67,180 respectively), as expected given the single allele correction in this heterozygous patient. For both RDEB01-Ks and RDEB05-Ks, C7 was detected in unedited control cells (Figure S11), making an analysis of gene repair based on protein expression challenging. However, for RDEB05-Ks, analysis by flow cytometry did indicate a modest increase in detectable C7 protein levels when plotted as a histogram of C7 mean fluorescence intensity (Figure S12).

Skin-derived fibroblasts express lower amounts of C7 compared with epidermal keratinocytes.^24^ In our flow cytometry assays, approximately 50% of a population of healthy donor fibroblasts seemed to be C7 positive due to the low levels of C7 expression and high levels of autofluorescence (Figure S13). Consequently, we were unable to obtain an accurate estimate of cellular C7 protein expression in relation to the estimated genomic *COL7A1* repair frequency. Nonetheless, our analysis demonstrated up to 14.5% C7 expression in bulk-edited RDEB03-Fs (Figure S13).

### HDR editing of RDEB-Ks restores expression and correct localization of C7 in bilayered SE models

**Figure 5:**
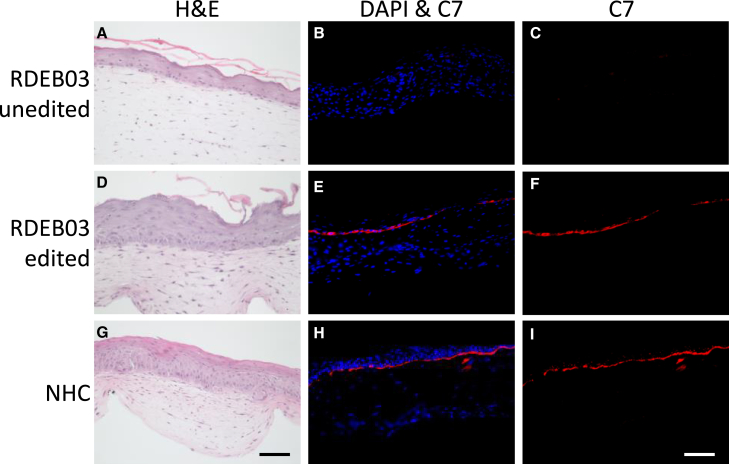
Edited RDEB cells demonstrate normal deposition of C7 to the BMZ in 3D SEs Representative images are shown from sections derived from 3D SEs (fibroblasts and keratinocytes) generated from unedited RDEB03 cells (A–C), edited RDEB03 cells (D–F) or NHCs (G–I). H&E staining is shown in (A, D, and G). Immunohistochemistry staining of C7 (red) and nuclei stained with DAPI (blue) is shown in (B, E, and H). C7 alone is shown in (C, F, and I). Scale bars, 100 μM.

We next assessed whether the high rates of HDR were sufficient to restore normal C7-expression in an *in vitro* 3D bilayered skin model—containing both fibroblasts and keratinocytes. Unedited and edited RDEB03 cells as well as normal human cells (NHCs) were used to generate SEs. Hematoxylin and eosin (H&E) histological analysis demonstrated normal skin architecture after five days of epidermal stratification (Figures 5A, 5D, and 5G). Immunohistochemistry analysis revealed no C7 in SEs generated using unedited RDEB03-cells (Figures 5B and 5C). In contrast, SEs generated from edited RDEB03-cells displayed a near-continuous deposition of C7 to the BMZ, comparable to SEs grown using NHCs (Figures 5E and 5F compared with Figures 5H and 5I). Further analysis of Ki67 expression, a keratinocyte proliferation marker, demonstrated the presence of Ki67 positive basal keratinocytes in gene edited SEs (Figure S14).

### Dual-nickase-mediated HDR is enhanced with M3814 but results in frequent large on-target deletions and modified transcription patterns

**Figure 6:**
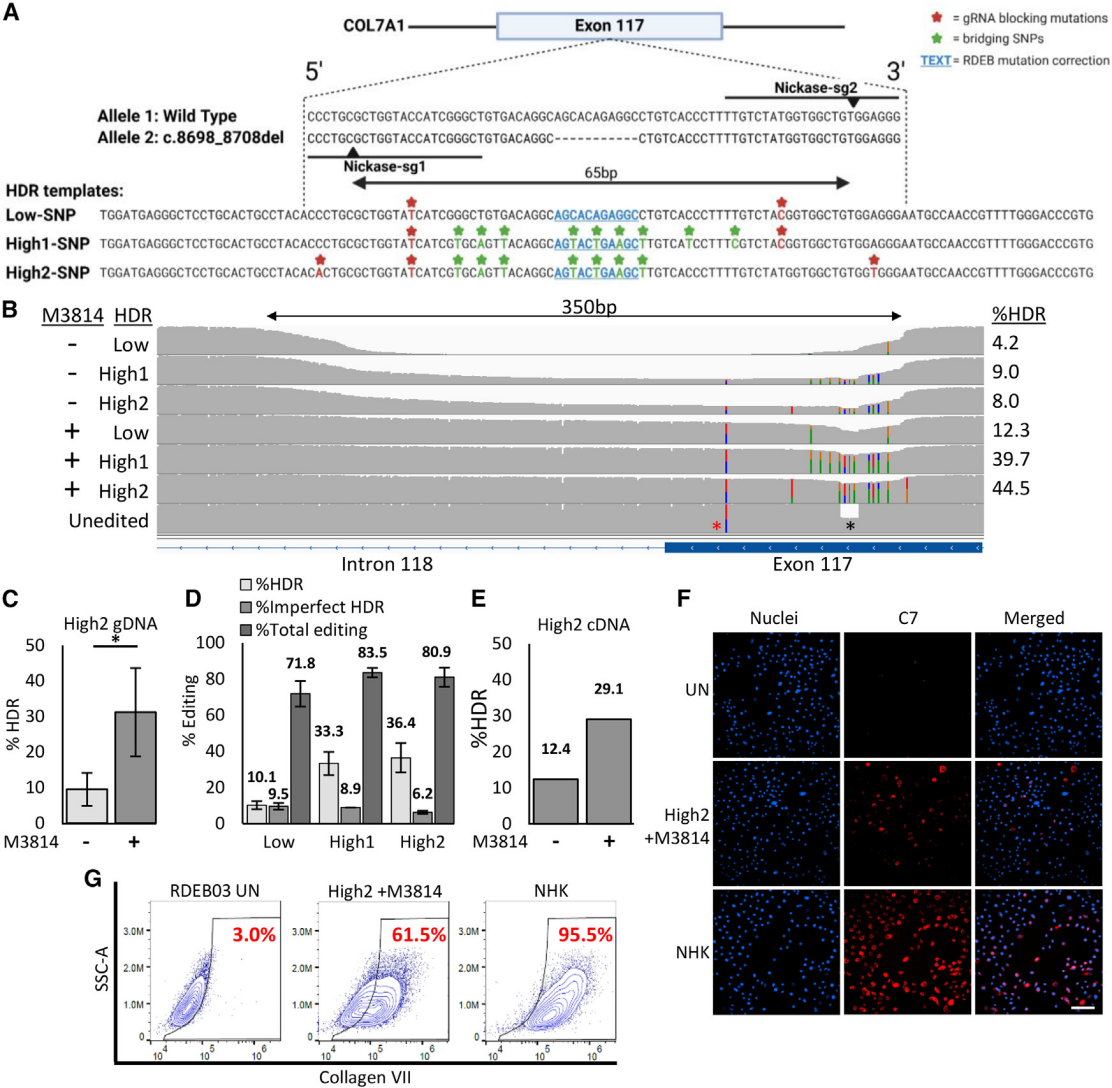
Evaluation of dual-nickase editing in RDEB03 keratinocytes (A) The dual-nickase based HDR-editing strategy for mutation c.8698_8708del present in RDEB03. Deleted bases are depicted with dashed lines. Black lines represent gRNA binding sites, with cut sites shown as black arrow heads. HDR templates are displayed below, showing mutation correction as underlined blue bases, gRNA blocking mutations as red bases (with asterisks), and bridging SNPs as green bases (with asterisks). (B) Representative ONT-seq data of dual-nickase RDEB03-Ks edited with or without M3814 as indicated, aligned to the reference genome and visualized using IGV. HDR templates and rates of genomic HDR, as assessed by ONT-Seq/Crispresso2 analysis, are shown. HDR-induced SNPs are indicated by colored lines on the IGV plots. A large 350-bp deletion is marked by a horizontal black arrow. The target 11 bp deletion (c.8698_8708del) is indicated with a black asterisk, and a non-target mutation (c.8780G>A) in *cis* with the WT allele is marked with a red asterisk. (C) Dual-nickase genomic HDR rates in bulk edited RDEB03-Ks edited with or without M3814, shown as mean ± SD (*n* = 3, *p* = 0.03). (D) Dual-nickase genomic HDR rates comparing three HDR templates with M3814. Percentages for perfect HDR (HDR), imperfect HDR, and total editing efficiency as shown (calculated as total HDR + indels, *n* = 2). (E) ONT-Seq/Crispresso2 analysis of cDNA derived from dual-nickase RDEB03-Ks edited with the High2-SNP template with or without M3814 (*n* = 1). (F) Immunocytochemistry analysis of C7 (red) in unedited (UN) and dual-nickase edited (edited) RDEB03-Ks and normal human keratinocytes (NHKs) as labeled. Nuclei are stained with DAPI (blue). Merged images are shown on the right-hand panel. Scale bar, 100 μM. (G) Flow cytometry analysis of C7 expression in unedited RDEB03-Ks (RDEB03 UN - left), RDEB03-Ks edited with High2-SNP with M3814 (middle), and NHKs (right). Plots show C7 on the x axis and side scatter (SSC-A) on the y axis. The percentage of C7-positive cells is indicated in red text.

Previous studies have demonstrated that editing with Cas9-nickase can reduce the genotoxic effects associated with Cas9-nuclease-induced DSBs.^25^^,^^26^ Therefore, we next aimed to enhance the specificity of editing by employing a dual-nickase strategy to correct the c.8698_8708del mutation in RDEB03-Ks. We designed two sgRNAs positioned in a PAM-out orientation, centered on the mutation, and spaced to create ssDNA nicks 65 bp apart (Figure 6A). Additionally, three HDR templates containing varying numbers of silent mutations (SNPs) were designed with the aim to restore the *COL7A1* reading frame while disrupting the sgRNA binding site to prevent further nicking (Figure 6A).

First, the impact of inhibiting DNA-PK on the dual-nicking strategy was investigated using M3814. RDEB03-Ks were co-electroporated with the two nickase-RNPs and one of the three HDR template variants. Analysis of an 870-bp PCR amplicon derived from genomic DNA spanning the target site revealed the presence of large deletions (approximately 350 bp) extending from the nickase-gRNA1 site into intron 118, present in 20%–90% of sequencing reads across all conditions (Figure 6B). In the presence of M3814, the frequency of these deletions decreased (Figures 6B and S15), and there was a significant increase in HDR rates, increasing from an average of 9.5% to 31.2% (average of all templates with or without M3814, *p* = 0.029) (Figures 6C and S16). Furthermore, higher HDR rates were obtained using the templates harboring a greater number of SNPs, averaging 33.3% and 36.4% for High1 and High2, respectively, compared with the low-SNP template at 10.1% (Figure 6D). Interestingly, 6.2%–9.5% of reads contained imperfect HDR events (Figure 6D), which was substantially higher than imperfect HDR events recorded when editing with Cas9 nuclease (at most 1.9%) (Figure S2D), indicating that the DNA repair mechanisms used during nickase gene editing may be error prone. The highest HDR rate achieved with the dual-nicking approach was 44.5% in low passage (P2) RDEB03-Ks edited with the High2-HDR template in the presence of M3814 (Figures 6B–6D).

Next, to assess the impact of gene editing at the mRNA level, *COL7A1* transcripts were isolated from dual-nickase-edited RDEB03-Ks, converted to cDNA and analyzed with ONT-seq. Consistent with our observations in Cas9-nuclease edited RDEB03-Ks (Figure 4A), we detected an increase in exon 117-skipped transcripts post editing, comprising of between 30% and 50% of transcripts in all nickase-edited RDEB03-Ks (Figure S17). Additionally, we observed aberrant splicing characterized by the adoption of a new splice acceptor site (AG) in up to 12% of transcripts from samples edited with the high-SNP template (Figure S17). Overall, in low passage (P2) RDEB03-Ks edited with the High2-SNP template, the proportion of transcripts with precise HDR in exon 117 was 12.4% without M3814 and 29.1% with M3814 treatment (Figure 6E).

Finally, the restoration of C7 expression in bulk-edited RDEB03-Ks was demonstrated by immunocytochemistry (Figure 6F). Flow cytometry analysis revealed that up to 61.5% of the bulk cell population was C7-positive (Figures 6G and S18), which was higher than the estimated 44% HDR correction rate indicated by ONT-seq (Figure 6B). This discrepancy is likely attributed to a combination of imperfect HDR events and indels that also contribute to C7 expression. Although C7 expression was restored, the level of indels was deemed unacceptably high, suggesting that Cas9 nuclease is a more suitable editing strategy for this locus.

## Discussion

Gene editing of autologous cells presents a promising avenue for the treatment of currently incurable genetic disorders. For RDEB, various gene therapy approaches, including gene replacement and gene-correction therapies are currently undergoing clinical trials.^3^ Notably, the U.S. Food and Drug Administration recently approved beremagene geperpavec (B-VEC, administered as VYJUVEK), a topical gene replacement therapy which utilizes a herpes simplex virus type 1 as a delivery vector for C7 to RDEB wounds.^27^ While beremagene geperpavec is highly effective for treating RDEB wounds, its therapeutic effects are temporary and necessitate repeated applications on newly formed or extensive wounds. A potentially more durable approach is the genetic correction of the endogenous *COL7A1* gene in patient-derived cells. While topical or systemic delivery of gene-editing reagents would be the preferred delivery method, the *ex vivo* approach currently offers higher correction efficiency and enables comprehensive safety evaluations before the genetically modified cells are reintroduced to the patient. For RDEB, engineered bilayered skin substitutes—generated using gene-corrected autologous keratinocytes (epidermis) and fibroblasts (dermis)—may offer an effective method for addressing cutaneous symptoms.^28^

Preclinical studies have suggested that achieving between 20% and 35% *COL7A1* correction of bulk-edited cells is sufficient for complete phenotypic reversion of RDEB.^9^^,^^10^ As such, several *ex vivo* gene-editing strategies have been investigated, with NHEJ- and HDR-mediated approaches being the most extensively studied due to their high efficiency and capacity to target a range of mutations.^3^^,^^29^ NHEJ-mediated repair directly rejoins cut DNA ends and is particularly effective for an exon skipping gene editing approach. Here, the mutation harboring exon is excised by creating two DSB in the flanking introns.^16^^,^^30^^,^^31^^,^^32^ This method holds therapeutic potential as the C7 protein contains a triple helical domain encoded by 84 in-frame exons.^16^ Many of these may be amenable to excision without compromising the functionality of the truncated protein.^16^ NHEJ-mediated repair may also be useful to target a dominant heterozygote mutation using a mutation specific guide. This approach has been used to target dominant mutations in keratin that cause EB simplex^33^^,^^34^ and once to target *COL7A1* in a dominant dystrophic EB study to our knowledge.^35^

While NHEJ-mediated repair is highly efficient, gene correction mediated by HDR may be preferred or necessary for correcting certain mutations, especially in regions where exon skipping is not suitable because the exon is essential for protein function. Therefore, developing methods that enable high rates of precise HDR-mediated repair is crucial for the broad application of HDR editing to any loci or mutation. This is particularly important given the diverse mutational landscape of *COL7A1*, with more than 650 mutations described for DEB (https://www.deb-central.org/).^36^^,^^37^ While some mutations, such as the *COL7A1* c.6527insC Spanish mutation, are more prevalent—constituting up to 46% of alleles in the Spanish RDEB population^6^^,^^30^^,^^31^^,^^32^—the mutational landscape in other regions can be more diverse or largely unknown due to the lack of population-scale genetic analysis of EB.^38^^,^^39^^,^^40^ To date, HDR-mediated gene editing research for RDEB has predominantly focused on homozygous *COL7A1* mutations.^6^^,^^7^^,^^9^^,^^30^^,^^31^^,^^32^ To our knowledge, only three studies have used HDR-mediated editing to address compound heterozygous recessive *COL7A1* mutations,^9^^,^^11^^,^^41^ and each achieved low HDR rates, necessitating positive enrichment or cell cloning to reach therapeutic levels.^11^^,^^41^ In our study, all three EB donors from New Zealand carry compound heterozygous recessive mutations, none of which have been targeted before for gene therapy, beyond our own *COL7A1* exon-skipping study.^16^ Therefore, we aimed to achieve highly efficient, high-fidelity repair of heterozygous *COL7A1* mutations using a straightforward HDR approach that can be readily applied to a broad range of EB-causing mutations.

Here, the CRISPR-Cas9 gene editing machinery was electroporated into cells as RNPs with ssDNA repair templates in the presence of the DNA-PK inhibitor M3814. M3814 suppresses NHEJ and promotes DNA repair via HDR.^20^ Our results demonstrated a nearly 3-fold increase in HDR rates in both primary RDEB fibroblasts and keratinocytes when M3814 was included in the editing process. In keratinocytes, HDR rates consistently exceeded 40% and reached up to 75%, as assessed over three separate *COL7A1* mutations. This translated to restored C7 expression in up to 80% of cells. Furthermore, gene-edited RDEB cells behaved similar to WT cells when incorporated into a 3D bilayered skin-equivalent model, with accurate deposition of C7 to the BMZ. To our knowledge, these results represent the highest, precise HDR-mediated gene editing rates reported to date in epidermal keratinocytes (75%).

Accurate analysis of CRISPR edits is essential for reliable reporting of gene-editing rates and ensuring the safety of these approaches. Traditional methods like PCR-band analysis via gel electrophoresis or Sanger sequencing of PCR amplicons often lack precision and are subject to biases introduced by PCR and sampling methods.^6^^,^^8^^,^^9^^,^^16^^,^^32^^,^^42^ These biases can lead to incorrect data representation, impacting the validity and reproducibility of editing results. Therefore, a robust pipeline for on-target gDNA editing analysis is crucial. We used ONT-seq for this purpose as this method delivers relatively inexpensive data, provides individual read resolution, and can be used to sequence long amplicons (>500 base pairs), enabling a more comprehensive detection of CRISPR edits. For instance, the nearly complete deletion of a 350-bp region in dual-nickase-edited samples would likely have gone undetected using standard Illumina sequencing due to the loss of a primer-binding site.^42^ However, while ONT-seq enhances the detection of larger indels, amplicon sequencing is still prone to PCR amplification bias and its efficacy is constrained by the length of the amplicons that can be produced. For a more thorough analysis, combining ONT-seq with methods designed to detect structural variants would be beneficial.^42^ For instance, recent studies have paired short-read sequencing for HDR-analysis^9^^,^^43^ with CAST-seq^44^ to identify kilobase-sized deletions, insertions, and chromosomal translocations in edited cells, which were missed by short-amplicon sequencing alone.^13^ Therefore, while our ONT-seq pipeline effectively identified larger indels, integrating additional methods like CAST-seq will be critical for the clinical translation of gene-editing techniques, especially when modulating repair pathways such as inhibiting NHEJ, which has been shown to increase the rate of MMEJ, large deletions and chromosomal translations and truncations.^45^^,^^46^^,^^47^ Furthermore, while outside the scope of this study, other methods such as CIRCLE-seq^48^ or spectral karyotyping^20^ could also be used for a more comprehensive off-target/genome-wide analysis. Additional assays that assess clonal outgrowth or effects on cell survival and proliferation would also be useful to assess unintended effects of off-target editing.

Recent studies have indicated that HDR templates which incorporate silent mutations in both the spacer (gRNA) sequence and PAM site can markedly increase HDR rates.^18^^,^^49^^,^^50^ We hypothesized that similar principles might apply to skin cells and could be useful for correcting *COL7A1* mutations. However, we found that for RDEB03 and RDEB05 derived cells, HDR templates with fewer SNPs achieved the highest HDR efficiency, demonstrating a more than 10% increase in editing rates as compared with high-SNP templates, although this increase was not significant. Interestingly, we observed an opposite effect when using a dual-nicking strategy for RDEB03 derived cells. Here templates with more SNPs improved HDR efficiency by up to 32%. However, it is important to note that DNA repair outcomes are complex and context dependent, influenced by factors such as target sequence, epigenetic landscape, donor-oligo design, and cell type.^49^^,^^51^ Therefore, it is unlikely that a universal HDR template design exists, so each unique application will require validation. Nonetheless, our results highlight that subtle changes in HDR template design can lead to potentially biologically significant differences in HDR efficiency and should be considered in future HDR-editing applications.

In this study, we focused primarily on RDEB donor 3 (RDEB03) due to their null C7 phenotype. The targeted mutation is in exon 117 of *COL7A1*, which contributes to the 30-kD NC2 domain of C7. This domain is crucial for the stability of the C7 protein and the aggregation of C7 molecules into functional anchoring fibrils. However, the protein region encoded by exon 117 is cleaved following dimerization of C7 homotrimers,^52^ suggesting that the function of exon 117 is primarily in the initial stability and folding of C7, rather than in AF formation or adherence at the BMZ. While not all mutations within the NC2 domain will lead to C7 instability, in cells derived from RDEB03, full-length mRNA is detected but no C7 protein is observed, indicating that the exon 117 mutation results in rapid degradation of the proto-C7 protein. Therefore, we predict that any stable C7 protein resulting from gene correction of exon 117 is likely to be functional.

Exon 117-skipped *COL7A1* mRNA transcripts were generated with both nuclease and nickase-mediated editing and so this skipping phenomenon appears to be agnostic to how the edits are generated. We observed a correlation between genomic correction, cellular expression, and the level of mRNA transcripts containing corrected exon 117 in Cas9 nuclease edited cells. Based on this, we hypothesize that transcripts missing this exon produce an unstable and rapidly degraded protein. This aligns with our hypothesis relating to the 11-bp deletion in this exon, but further research is needed to confirm this and to understand what mechanisms underlie exon 117 skipping.^53^

We also observed an additional HDR template-dependent effect on mRNA splicing within exon 117, specifically induced by templates with a high number of SNPs and absent in templates which only introduced an SNP at the PAM site. This splicing effect generated transcripts missing the first 120 of the 197 nucleotides normally present in exon 117, with no impact on the splicing of downstream intron 118. Therefore, although templated SNPs may enhance editing in some settings, our findings demonstrate that they should be introduced conservatively as editing outcomes may be hard to accurately predict. Furthermore, they underscore the necessity of robust post-editing analysis, including mRNA transcript and splicing analysis, to ensure efficacy and safety.

Finally, in an attempt to improve the specificity of our approach, we applied a dual-nickase strategy for correcting the c8698_8708del mutation, achieving up to 45% HDR in the presence of M3148. However, this method resulted in a high incidence of indels and large deletions (>350 bp), leading to stable but unexpected protein expression in standard 2D culture conditions. These deletions may have affected the splicing of the intron 117/exon 118 region, potentially causing the use of a delayed termination codon, although further analysis is required to confirm this possibility. Previous studies have also demonstrated that nickase mediated editing can lead to deletions ranging from hundreds of base pairs to kilobases due to MMEJ repair.^13^^,^^54^ Similar to NHEJ, MMEJ can be inhibited using small molecules targeting key repair proteins such as DNA-polymerase theta (POLθ) and replication protein A.^55^ Inhibiting both NHEJ and MMEJ simultaneously has been shown to enhance HDR more effectively than inhibiting either pathway alone.^56^ Therefore, combining POLθ inhibitors with M3814 in paired-nickase editing approaches could potentially reduce large deletions. Nonetheless, we concluded that a Cas9 nuclease strategy, which achieved higher rates of HDR with fewer large on-target aberrations was more suitable for this locus.

Overall, this study demonstrates a highly efficient method for *ex vivo* HDR editing in primary human skin cells and introduces a simple, cost-effective and robust pipeline for on-target editing analysis. We successfully targeted three heterozygous *COL7A1* mutations present in primary keratinocytes derived from three individual RDEB donors. Therapeutic HDR rates exceeding 40% were achieved in each case, with minimal optimization required for each separate locus. These data suggest that our editing pipeline can be easily adapted to target patient-specific mutations in other regions of *COL7A1* or used to edit other genes involved in a range of other EB subtypes and dermatological disorders. These methods therefore offer a promising avenue of treatment for people with EB.

## Materials and methods

### Design of sgRNAs and ssODN (donor) templates

sgRNAs were designed using the CHOPCHOP webtool (https://chopchop.cbu.uib.no/) and selected based on their proximity to the mutation, predicted efficiency and number of off-target sites (Tables S1 and S2). To ensure the top predicted off-target sites were correct, the gRNAs were also checked using the IDT CRISPR-Cas9 guide-RNA design checker (https://sg.idtdna.com/site/order/designtool/index/CRISPR_CUSTOM) and the POP-off bioinformatic pipeline to account for potential frequent population variants.^57^ Short ssODN templates (donor templates) (Table S3) were designed manually, with symmetrical approximately 35 nucleotide homology arms extending from the final SNP. We note that low-SNP and med-SNP templates were kept at a consistent length to the high-SNP templates to enable comparison. Care was taken to ensure that the codons generated by the SNPs were used in a similar frequency to the original codon, so as not to disrupt standard translation and processing of the protein.

### Cell culture and nucleofection of primary RDEB skin cells

Primary human cells (keratinocytes and fibroblasts) were isolated from human skin tissue after enzymatic digestion of whole human skin as described previously.^58^^,^^59^ RDEB cells were obtained from skin biopsies from fully consenting adult donors under ethics 19/STH/47 provided by the Southern Health and Disability Ethics Committee. Healthy human tissue was donated by patients undergoing elective surgeries, including breast reduction, reconstruction, and abdominoplasty, and was approved by the New Zealand Northern Health and Disability Ethics Committee (approval number NTX/08/09/086).

Primary dermal fibroblasts were cultured in DMEM (Gibco, Thermo Fisher Scientific, Waltham, MA, USA) supplemented with 10% fetal bovine serum (FBS) (Moregate Biotech, Bulimba, QLD, Australia) and 100 U/mL PS (Gibco). Epidermal keratinocytes were cultured in a modified Kelch’s medium^58^ as follows: DMEM without calcium and glutamax (Gibco), F12 (Gibco) (3:1 DMEM:F12), 10% FBS (Moregate), 1× Glutamax (Gibco), 20 ng/μL KGF (Peprotech, Rocky Hill, NJ, USA), 0.625 μg/mL amphotericin B, 100 U/mL PS (Gibco), 0.4 μM SB 772077B (Tocris Bioscience, Bristol, UK). Keratinocytes were seeded at between 4 × 10^3^ and 2 × 10^4^ cells/cm^2^ (depending on the required timing) and maintained on a bed of irradiated (50 Gy) 3T3-J2 murine fibroblast feeder cells (2 × 10^4^ cells/cm^2^) (Kerafast, Boston, MA, USA). At each passage, the 3T3-J2 feeder cells were lifted and discarded by washing in 0.5 M EDTA/PBS (Gibco) for 5 min at 37°C. Keratinocytes were then detached with TrypLE (Gibco). All cells were maintained at 37°C and 5% carbon dioxide in a humidified incubator.

Electroporation was performed using the Amaxa 4D Nucleofector (Lonza Bioscience, Basel, Switzerland). RNPs were complexed by combining 75 pM sgRNA (Integrated DNA Technologies, IDT, Coralville, IA, USA) and 15 pM Cas9 HiFi nuclease (IDT) or Cas9 D10 nickase (IDT) (5:1 sgRNA ratio) at room temperature (RT) for 20 min immediately before nucleofection. For fibroblasts, 1 × 10^5^ cells were electroporated using the P2 nucleofection kit (Lonza Bioscience, pulse code DT-130) with RNPs and 50 pM of ssODN (IDT). For keratinocytes, 3 × 10^5^ cells were electroporated with the P3 nucleofector kit (Lonza Bioscience, pulse code CM-137) with RNPs and 50 pM ssODN (IDT). After electroporation, cells were left for 15 min at RT before being transferred to pre-equilibrated medium. A complete medium change was conducted 24 h post-editing. M3814 (MedChemExpress, Monmouth Junction, NJ, USA) was added to pre-equilibrated medium to a final concentration of 1 μM and was retained for 72 h after editing.

### ONT-seq of bulk-edited DNA and cDNA

Genomic DNA was extracted from 1–5 × 10^5^ bulk-edited cells using the Monarch Genomic DNA Purification Kit (New England Biolabs [NEB], Ipswich, MA, USA). Total mRNA was extracted from 5 × 10^5^ bulk-edited cells using the RNAqueous Total RNA Isolation Kit (Thermo Fisher Scientific, Waltham, MA, USA) and converted to complementary DNA (cDNA) with the iScript cDNA Synthesis Kit (BIO-RAD, Hercules, CA, USA). Genomic DNA and cDNA were amplified with Phusion High-Fidelity DNA Polymerase (NEB) following the recommended cycle settings (https://tmcalculator.neb.com/#!/main) based on the primer pair used (IDT) (Table S3). For ONT-seq, 200 fM of each amplicon were input into the Ligation Sequencing V14 – PCR Barcoding (LSK-114 with EXP-PBC001) protocol and sequenced on the Flongle Flow Cell (R10.4.1) for 24 h (ONT, Oxford, UK). An average of 21,752 aligned reads were achieved across all sequenced samples (Figure S19). In all sequencing runs, an unedited control for each sequenced loci was included to account for ONT-seq errors. Additionally, cDNA from healthy donors was included for each cell type as appropriate RNA splicing controls.

Base calling was performed using the Guppy Base caller (ONT, Super accurate model), and the base-called reads were filtered for full-length reads (primer to primer sequences) and trimmed to remove barcode and adapter sequences using SAMtools. For analysis of genomic DNA, the cleaned fastq files were aligned to the human reference genome (GRCh38) with MiniMap2, and BAM files were visualized with Integrative Genomics Viewer (IGV). Alternatively, the cleaned fastq files were submitted to CRISPResso2 for standard analysis. For cDNA, the cleaned fastq files were aligned to the human reference genome using MiniMap2 with splice awareness and visualized with IGV. All code is available on request.

### Estimation of HDR-mediated cellular correction using CRISPResso2 analysis

HDR and indel rates were calculated and reported as estimates of the frequency of cellular correction. For allele-specific gRNAs, editing was reported as a percentage of the targeted MUT allele, which constituted 50% of the total reads (comprising MUT and WT) alleles). For example, if HDR was observed in 25% of total reads, this corresponds with 50% HDR attributable to the MUT allele (25%/50% MUT reads) and equates to an estimated 50% cellular correction. For non-allele specific gRNAs (including nickase guides), the editing rate is a percentage of total reads. In these cases, an equal distribution of editing events between alleles was either calculated (Figure 2H) or assumed. For instance, if 60% of total reads demonstrate HDR, this equates to 30% HDR attributable to the MUT allele (30%/50%) and, therefore, an estimated 60% cellular correction.

### Immunocytochemistry of primary keratinocytes

For immunocytochemistry analysis, 10,000 keratinocytes were seeded into an eight-well chamber slide (Nunc, Thermo Fisher Scientific) under standard culture conditions. After 72 h, the 3T3-J2 feeder cells were removed using standard protocols, and the remaining keratinocytes were washed twice with TBS and then fixed with a 4% formaldehyde solution (Thermo Fisher Scientific) for 10 min at RT. Permeabilization was performed using 0.5% Triton X-100 (Sigma-Aldrich, St. Louis, MO, USA) for 10 min at RT. Cells were then blocked with 0.25% Casein in TBS containing 10% human serum (Thermo Fisher Scientific) for 30 min. For the primary antibody co-stains, a human-specific mouse IgG1 anti-C7 antibody (LH7.2, Invitrogen) at a 1:200 dilution and a rabbit anti-FOXM1 antibody (D3F2B, Cell Signaling, NEB) at a 1:200 dilution were prepared in TBS with 10% human serum. Staining was carried out overnight at 4°C. After three 5-min washes with TBS at RT, cells were co-stained with Alexa Fluor 647 goat anti-mouse IgG1 (1:200) and Alexa Fluor 555 goat anti-rabbit (1:1,000) secondary antibodies (Molecular Probes, Invitrogen), along with 2.5 μg/mL DAPI, for 1 h at RT. Finally, after two 15-min washes with TBS at RT, coverslips were mounted using ProLong Gold (Invitrogen) mounting medium. Imaging was performed using a Nikon Ni-U Upright microscope or Andor Revolution microscope and analyzed with FIJI-ImageJ.

### Flow cytometry analysis

Collagen VII expression in fibroblasts and keratinocytes was detected via flow cytometry. For each sample, 5 × 10^5^ cells were labeled using the Zombie NIR Fixable Viability Kit (Biolegend, San Deigo, CA, USA) and then fixed and permeabilized using the eBioscience Foxp3/transcription factor staining buffer set (Thermo Fisher Scientific) according to the manufacturer’s instructions. Samples were washed with permeabilization buffer and resuspended in permeabilization buffer supplemented with 10% v/v goat serum (Gibco) and 8 μg/mL anti-human-collagen VII clone LH7.2 (Invitrogen) overnight at 4°C. After another wash with permeabilization buffer, samples were incubated in permeabilization buffer supplemented with 1 μg/mL goat anti-mouse IgG1-Alexa Fluor 488 at RT for 60 min. Unstained cells, cells labeled only with Zombie Near-IR, and cells labeled with Zombie Near-IR and incubated with secondary antibody in the absence of LH7.2 were used as negative controls. Fibroblasts and keratinocytes from healthy donors were included as positive expression controls for Collagen VII. Samples were acquired on a Cytek Aurora flow cytometer and analyzed using FlowJo Software vX (BD Biosciences). Flow cytometry acquisition was performed at the Auckland Cytometry Shared Research Equipment Center, School of Biological Sciences, University of Auckland.

### Generation of 3D bilayered SEs and histological/immunofluorescence analysis

We constructed 3D bilayered SEs using the methods described in detail,^60^ with slight modifications. Briefly, SEs were generated by seeding 1 × 10^5^ fibroblasts into a 3 mg/mL bovine collagen I gel containing, collagen (Organogenesis, Canton, MA, USA), 1× MEM (Thermo Fisher Scientific), GlutaMAX (Thermo Fisher Scientific), 10% FBS (Moregate), and sodium bicarbonate (Sigma-Aldrich). The collagen gels were set in Transwell inserts (Organogenesis). The SEs were then submerged in DMEM (Gibco) with 10% FBS (Moregate) and allowed to contract by incubation at 37°C for 1 week. Meanwhile, keratinocytes were prepared by culturing in standard growth medium containing only 5% FBS for at least one passage. After 1 week, all the medium was aspirated from the SEs. Keratinocytes were split following standard protocols and resuspended in epidermalization medium 1 (EPI1), which includes DMEM without calcium and GlutaMAX (Gibco), F12 Gibco (3:1 DMEM:F12), 4 mM L-glutamine (Gibco), 40 μM adenine (Sigma-Aldrich), 1 μM hydrocortisone (Sigma-Aldrich), 20 nM tri-iodothyronine (T3) (Sigma-Aldrich), 10 μg/mL insulin (Sigma-Aldrich), 2 nM progesterone (Sigma-Aldrich), 0.1% FBS (Moregate), 0.4 μM SB 772077B, and 20 ng/μL KGF. Carefully, 5 × 10^5^ keratinocytes were seeded on top of the SEs and left for 1 h at 37°C to adhere. SEs were submerged in EPI1 and incubated at 37°C for 72 h. Next, EPI1 was completely aspirated and replaced with EPI2 medium (same as EPI1 with the addition of 1.8 mM calcium chloride [Sigma-Aldrich]). SEs were then incubated at 37°C for 5 days, with a complete medium change approximately every 48 h. EPI2 was then aspirated completely, and the SEs were placed at an air-liquid interface in cornification medium (DMEM without calcium and GlutaMAX, F12 [1:1 DMEM:F12 ratio], 4 mM L-glutamine, 40 μM adenine, 1 μM hydrocortisone, 20 nM triiodothyronine, 10 μg/mL insulin, 2% FBS, and 1.8 mM calcium chloride). SEs were incubated at an air-liquid interface in cornification medium for 1 week, with complete medium changes every 48 h. SEs were then harvested by cutting each SE in half. One-half was processed for paraffin embedding, while the other one-half was embedded in frozen OCT tissue as described previously.^60^

For H&E analysis, paraffin-embedded tissues were sectioned to 5 μM using a Microtome and were stained using standard H&E protocols. Sections were analyzed by standard brightfield microscopy at 20× magnification on a Nikon Eclipse 80i upright microscope. For immunohistochemistry, OCT blocks were sectioned to 5 μM with a Leica Cryotome (CM1860UV). Sections were fixed with 4% formaldehyde solution for 10 min at RT and then permeabilized in 0.5% Triton X-100 for 10 min at RT. Cells were then blocked with 0.25% casein in TBS containing 10% human serum for 30 min. For the primary antibody co-stains, a human-specific mouse IgG1 anti-C7 antibody (LH7.2, Invitrogen) at a 1:200 dilution and a rabbit anti-Vimentin (EPR3776, Abcam) or a rabbit anti-P63 (4A4, Abcam) antibody were prepared in TBS with 10% human serum. Staining was carried out overnight at 4°C. After three 5-min washes with TBS at RT, cells were co-stained with Alexa Fluor 488 goat anti-mouse IgG1 (1:200) and Alexa Fluor 555 goat anti-rabbit (1:1,000) secondary antibodies (Molecular Probes, Invitrogen), along with 2.5 μg/mL DAPI, for 1 h at RT. Finally, after two 15-min washes with TBS at RT, coverslips were mounted using ProLong Gold (Invitrogen) mounting medium. Imaging was performed using a Nikon Eclipse 80i microscope and analyzed with FIJI-ImageJ.

## Data availability

The full datasets generated and analyzed during the current study are available from the corresponding author on request.

## Acknowledgments

We acknowledge the individuals with EB who generously donated skin samples for this research. We thank Auckland Genomics for their assistance in developing ONT sequencing and subsequent analysis pipelines, and for conducting Sanger sequencing. We are grateful to Chun-Jen Jennifer Chen for providing assistance with skin tissue preparation and histology methods. We also acknowledge Professor Johnathan Garlick’s research team, namely Sasha Shenk and Isha Singh, for their assistance and shared expertise in methods related to 3D skin equivalent models and cell culture.

We thank the Auckland Medical Research Fund (grant number 1120018), the Faculty of Science at the 10.13039/501100001537University of Auckland (grant number 3717415), the School of Biological Sciences at the 10.13039/501100001537University of Auckland, and the Maurice Wilkins Center for funding this study.

## Author contributions

J.H. conceptualization (support), data curation and analysis (lead), investigation (lead), methodology (lead), writing –original draft (lead). A.d.R. investigation (supporting), writing – review and editing. D.V. investigation (supporting), data visualization (supporting), writing – review and editing. L.C. methodology (supporting), writing – review and editing. E.L. investigation (supporting). C.M. methodology (supporting). B.B. methodology (supporting). D.K. methodology (supporting). Y.M. methodology (supporting). J.G methodology (supporting), resources (supporting). PR.D. resources (supporting). D.P. resources (supporting). V.F. methodology (supporting), writing – review and editing. H.S. conceptualization (lead), funding acquisition (lead), project administration (lead), resources (lead), supervision (lead), writing – original draft (supporting), writing – review and editing (lead).

## Declaration of interests

The authors declare no conflict of interests.

## Supplemental information

Docment S1. Figures S1–S19 and Tables S1–S3

Document S2. Article plus supplemental information
