# Supplementary material for: Enhanced HDR-mediated correction of heterozygous COL7A1 mutations for recessive dystrophic epidermolysis bullosa
Source: Mol Ther Nucleic Acids. 2025 Feb 1;36(1):102472. doi: 10.1016/j.omtn.2025.102472 (PMC11872078; doi:10.1016/j.omtn.2025.102472)
Supplement: Docment S1. Figures S1–S19 and Tables S1–S3 [file mmc1.pdf]

OMTN, Volume *36*

Supplemental information

Enhanced HDR-mediated correction of heterozygous *COL7A1* mutations for recessive dystrophic epidermolysis bullosa

John M.T. Hunt, Alex du Rand, Daniel Verdon, Leah Clemance, Evert Loef, Chloe Malhi, Ben Buttle, David J.H.F. Knapp, Yale S. Michaels, Jonathan Garlick, P. Rod Dunbar, Diana Purvis, Vaughan Feisst, and Hilary Sheppard

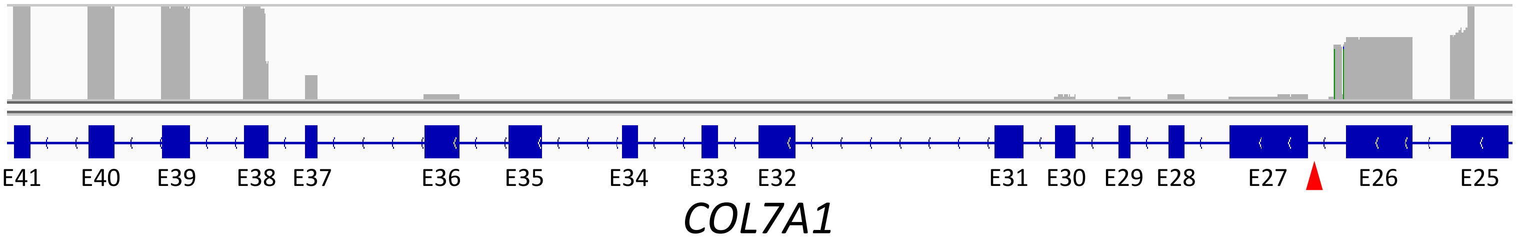

Figure S1: The RDEB05 splice site mutation, c.3551-3T>G, results in the sequential skipping of *COL7A1* exons 27 to 37. *COL7A1* transcripts were analyzed by ONT-sequencing and aligned to the reference genome, displayed as a coverage track in Integrative Genomics Viewer (IGV). The blue boxes at the bottom indicate *COL7A1* exons as labeled, and introns are marked as dashed blue lines. The red arrow indicates the location of the c.3551-3T>G mutation.

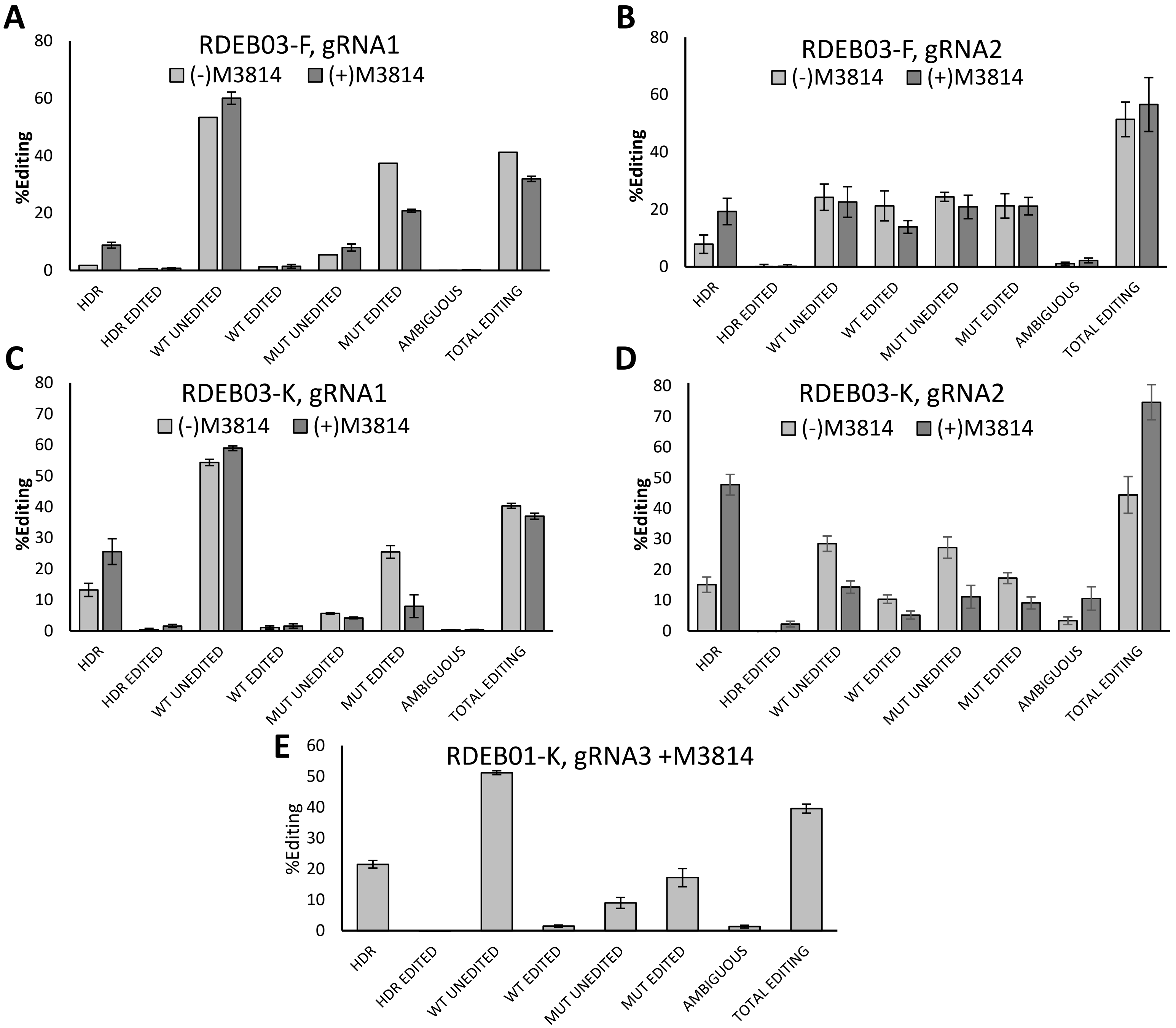

Figure S2: Editing with M3814 enhances HDR efficiency in nuclease RDEB03 fibroblasts and keratinocytes. (A-D) ONT-sequencing data are shown for RDEB03 fibroblasts and keratinocytes edited with either gRNA1 or gRNA2, the high-SNP HDR template, with or without M3814. Data is shown as allele frequencies reported from CRISPResso2 analysis. Left to right: percentage HDR, HDR edited, wild-type (WT) unedited, WT edited, mutation (MUT) unedited, MUT edited and ambiguous. MUT refers to the allele containing the targeted mutation (c.8698_8708del), while WT denotes the opposite allele. (A) RDEB03 fibroblasts edited with gRNA1 with (*n=1*) and without (*n=2*) M3814. (B) RDEB03 fibroblasts edited with gRNA2 with (*n=2*) and without M3814 (*n=2*). (C) RDEB03 keratinocytes edited with gRNA1 with (*n=3*) and without (*n=3*) M3814. (D) RDEB03 keratinocytes edited with gRNA2 with (*n=3*) and without M3814 (*n=3*). (E) RDEB01 keratinocytes edited with gRNA3 with M3814 (*n=3*, compiled across different HDR templates).

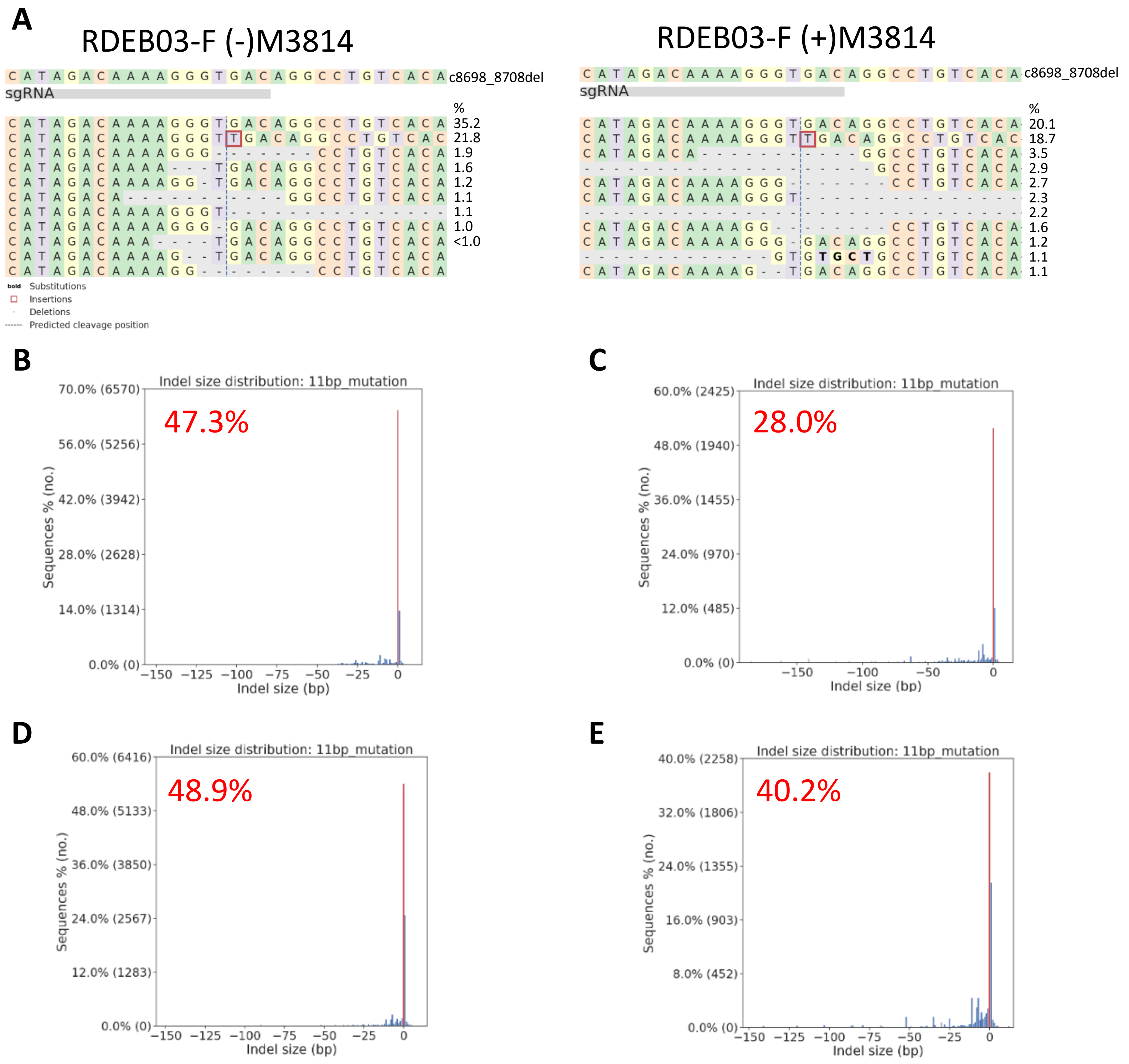

Figure S3: Editing with M3814 results in a small increase in medium size deletions. (A) Screen capture of CRISPResso2 allele alignment showing indel distribution in RDEB03 fibroblasts edited with RDEB03 gRNA2 and high-SNP template. This alignment only includes reads classified as MUT-unedited and MUT-edited (see Figure 2). The reference allele is shown at the top (c.8698_8708del), with indels below in decreasing frequency (shown to <1%). The gRNA cut site is marked by a vertical dashed black line. Substitutions are in bold, insertions are outlined in red, and deletions are marked as dots. (B-E) Indel distribution plots from CRISPResso2 analysis of RDEB03 keratinocytes and fibroblasts edited with and without M3814. The x-axis shows indel size and the y-axis represents the percentage of the sequences with that sized indel. Note the y-axis indicates the percentage of reads within those classified as MUT-unedited or MUT-edited, with the total percentage of the c.8698_8708del reads shown in red. (B-C) RDEB03 keratinocytes edited without (B) and with (C) M3814. (D-E) RDEB03 fibroblasts edited without (D) and with (E) M3814.

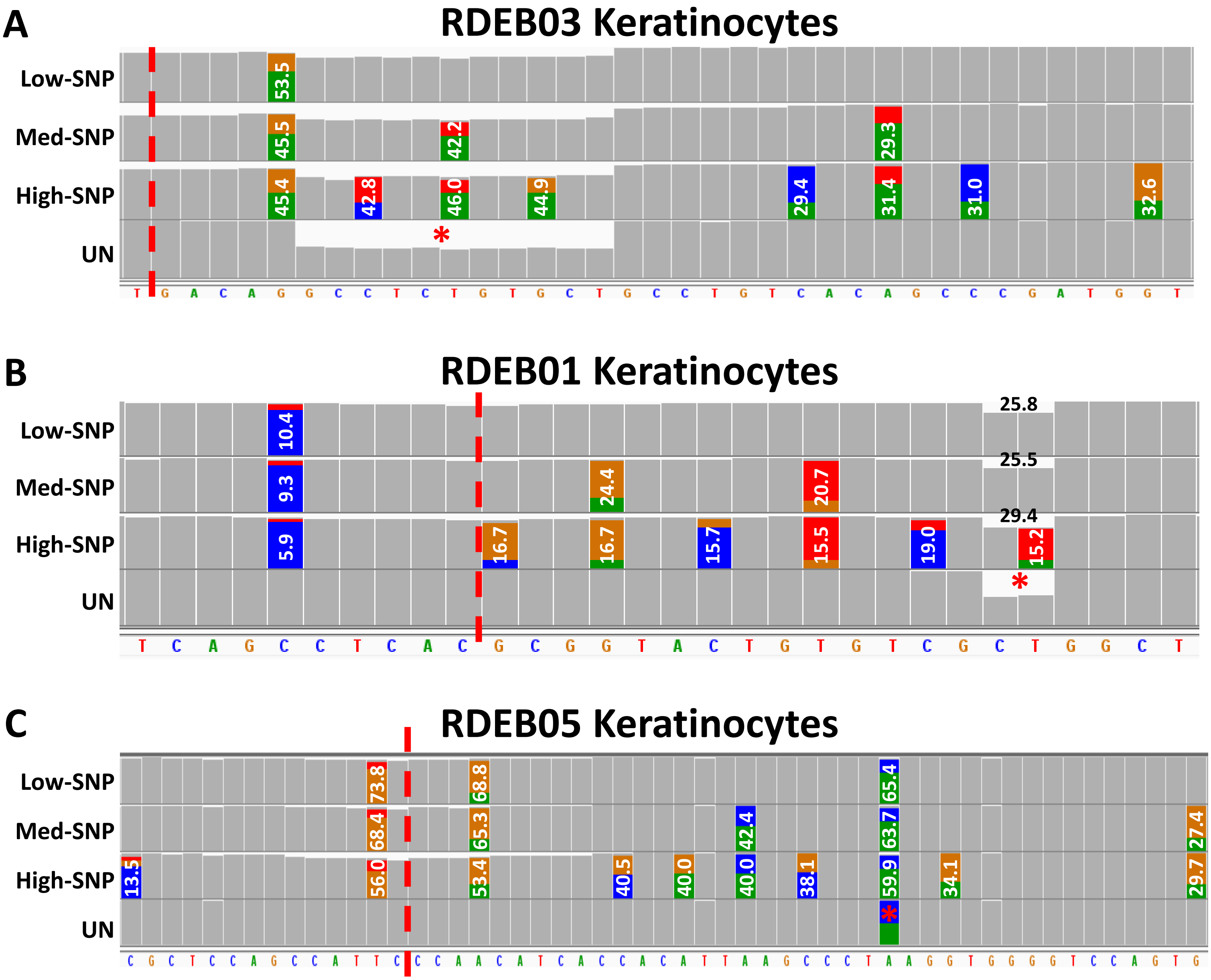

Figure S4: SNP incorporation rates when editing with HDR templates containing different numbers of SNPs for three RDEB donors. (A-C) Representative ONT-sequencing data displayed as coverage tracks in Integrative Genomics Viewer (IGV). The reference sequence is shown at the bottom of each diagram. HDR-induced silent mutations are colored (adenine – green, cytosine – blue, thymine – red, guanine – orange) with their frequencies indicated in white text on each SNP. The Cas9 cut sites are marked by a dashed red line. A red asterisk indicates each mutation. (A) RDEB03 keratinocytes edited with gRNA2 and various HDR templates in the presence of M3814. (B) RDEB01 keratinocytes edited with gRNA1 and various HDR templates in the presence of M3814. Black text above the mutation indicates the percentage of mutation remaining. (C) RDEB05 keratinocytes edited with gRNA1 and various HDR templates in the presence of M3814.

A

Name	OFT1			OFT2			OFT3			OFT4		
	%indel	%indel comp	Read#	%indel	%indel comp	Read#	%indel	%indel comp	Read#	%indel	%indel comp	Read#
**RDEB Unedited**	2.35	0	19057	2.39	0	15494	2.58	0	23604	2.45	0	22444
**Edited – M3814**	2.42	0.07	23602	2.41	0.02	19557	2.78	0.2	24664	2.49	0.04	17209
**Edited +M3814**	2.66	0.31	16925	2.31	0	11949	2.83	0.25	35595	2.80	0.35	14334

B

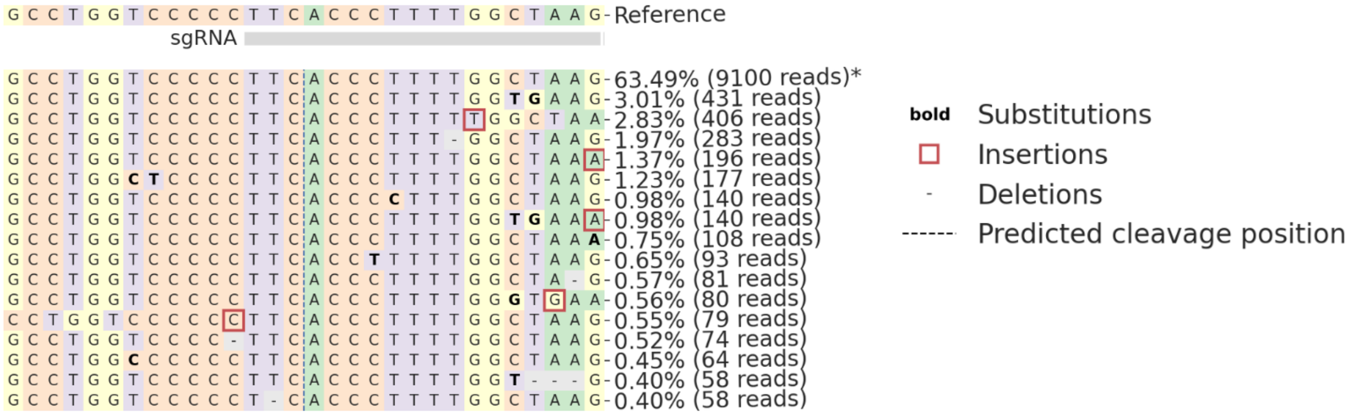

Figure S5: Nanopore Sequencing Analysis of Top Predicted Off-Target Sites. Low-passage (P2) RDEB03 keratinocytes were edited using gRNA2 and the Low-SNP HDR template, with or without M3814. Genomic DNA was analyzed for off-target editing by PCR amplification and sequencing of the top four predicted off-target sites. (A) The table presents the percentage of indels reported by ONT-seq/CRISPResso2 analysis of amplicons. Abbreviations include off-target loci 1-4 (OFT1-4), percentage indels (%indels), percentage indels compared to the unedited control (%indel comp), and read number (Read#). The indel composition of the cell highlighted in red is detailed in (B). (B) A screen capture from CRISPResso2 shows the allele alignment for the indel composition. The reference allele is at the top, with indels below in decreasing frequency. The gRNA cut site is marked by a vertical dashed black line. Substitutions are in bold, insertions are outlined in red, and deletions are indicated by dots.

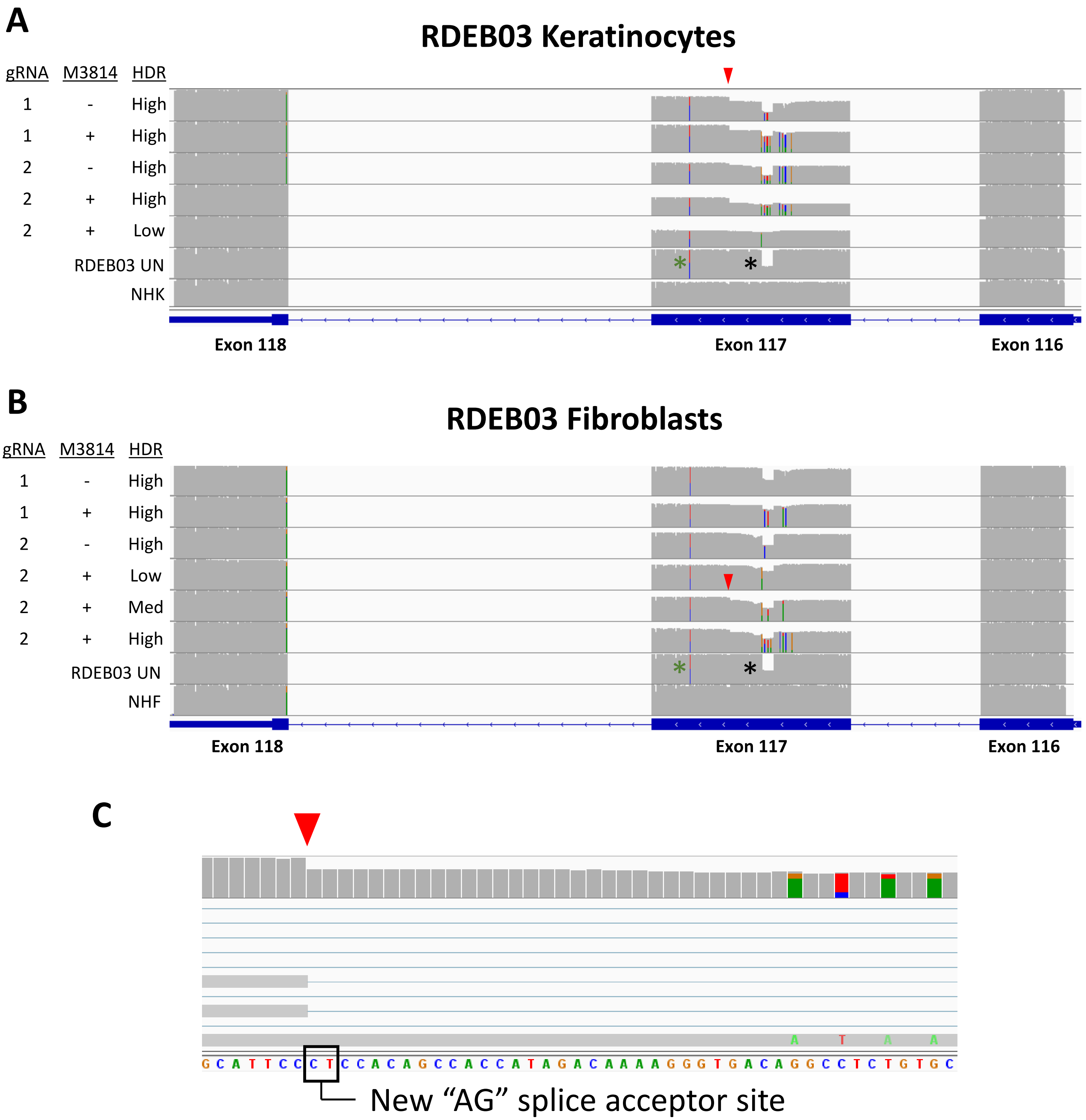

Figure S6: Analysis of *COL7A1* transcripts in nuclease edited RDEB03 keratinocytes and fibroblasts reveals modified splicing of exon 117. Various edited RDEB03 keratinocyte (A) and fibroblast (B). ONT-seq was performed on PCR-amplified cDNA prepared from mRNA, and alignments displayed as coverage tracks in IGV. (A-B) The sample conditions are listed on the left, where RDEB03 unedited (UN) and normal human keratinocytes (A, NHK) or normal human fibroblasts (B, NHF) act as control sequences. The target 11bp deletion (c.8698_8708del) is indicated with a black asterisk, the green asterisk indicates the other, non-target mutation (c.8780G>A) on the opposite allele. The location of an aberrant splice site is marked by a red arrow. (C) Magnification of the aberrant splice site reveals splicing is occurring at a new “AG” splice acceptor site, marked by a black square.

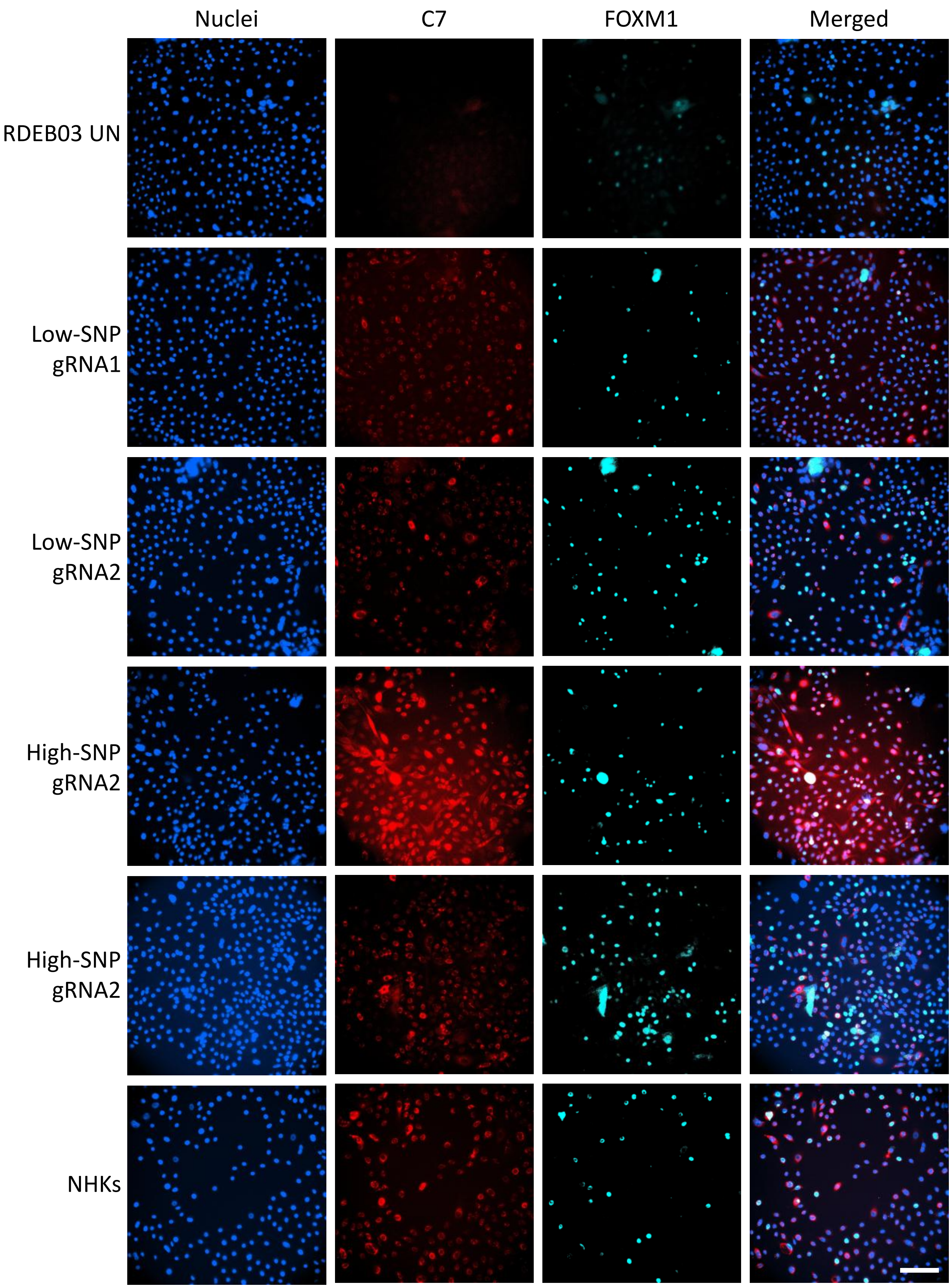

Figure S7: Restoration of C7 expression in gene edited RDEB03 keratinocytes. Immunocytochemistry analysis of C7 (red) in unedited (UN) and edited RDEB03-Ks (rows 2-5) and normal human keratinocytes (NHK) as labelled. Nuclei are stained with DAPI (blue). Nuclear FOXM1 staining (cyan) indicates progenitor cells. Merged images are shown on the right-hand side, where cyan represents merged DAPI (blue) and FOXM1 (cyan) nuclear stains. Scale bar (bottom right) represents 100µM. Conditions top to bottom: RDEB03 unedited, RDEB03 edited with gRNA1 and Low-SNP template, gRNA2 and low-SNP template, gRNA2 and high-SNP template (duplicated), NHKs. All samples were edited in the presence of M3814.

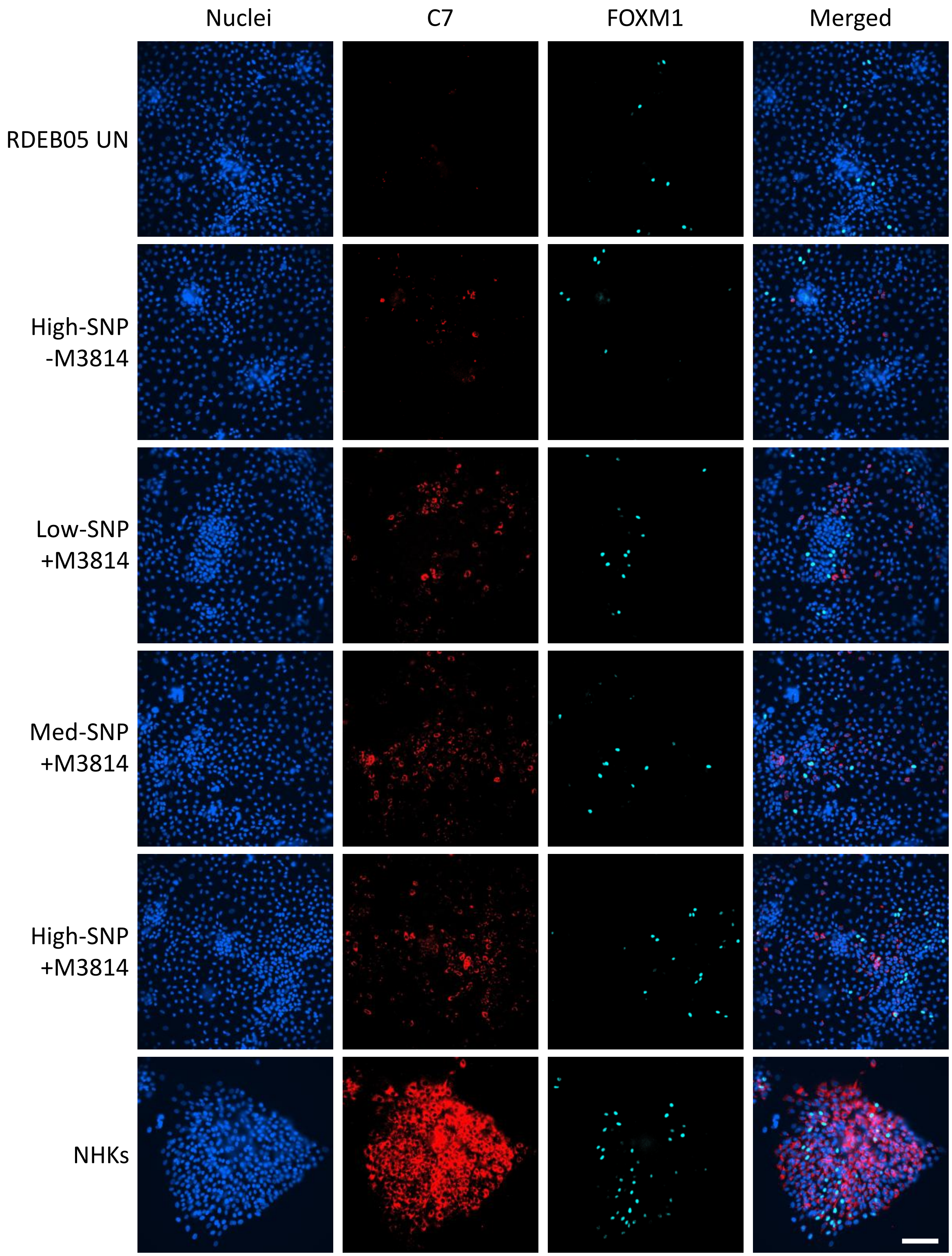

Figure S8: Restoration of C7 expression in gene edited RDEB05 keratinocytes. Immunocytochemistry analysis of C7 (red) in unedited (UN) and edited RDEB05-Ks (rows 2-5) and normal human keratinocytes (NHK) as labelled. Nuclei are stained with DAPI (blue). Nuclear FOXM1 staining (cyan) indicates progenitor cells. Merged images are shown on the right-hand side, where cyan represents merged DAPI (blue) and FOXM1 (cyan) nuclear stains. Scale bar (bottom right) represents 100µM. Conditions top to bottom: RDEB05 unedited, RDEB05 edited with the high-SNP template and M3814, low-SNP template with M3814, med-SNP template with M3814, high-SNP template with M3814, NHKs.

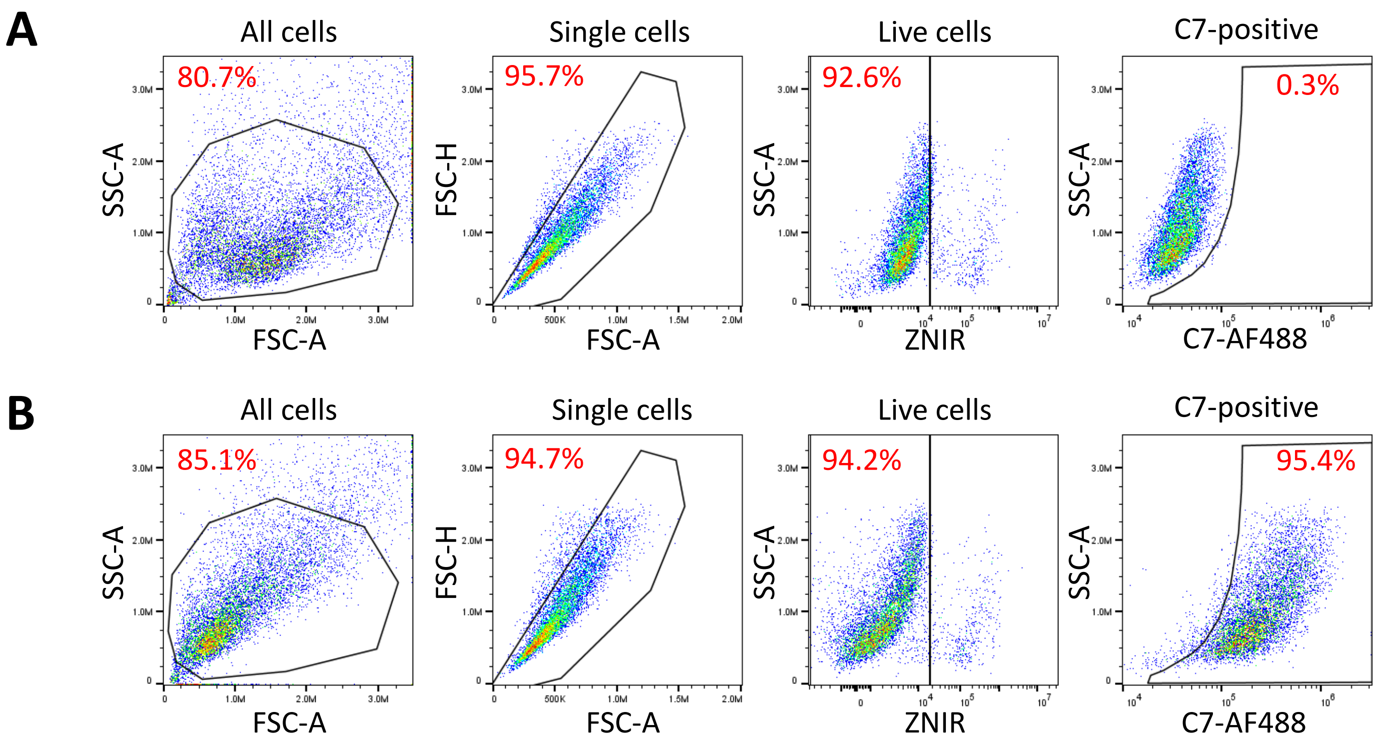

Figure S9: Flow cytometry scatterplots illustrating representative gating strategies for the detection of C7. From left to right: All cells, single cells, live cells (zombie-NIR viability stain), C7-positive cells (Alexa Fluor 488). Percentages for each group are indicated in red. Gating strategy is depicted for (A) secondary-only control and (B) normal human keratinocyte full stain control.

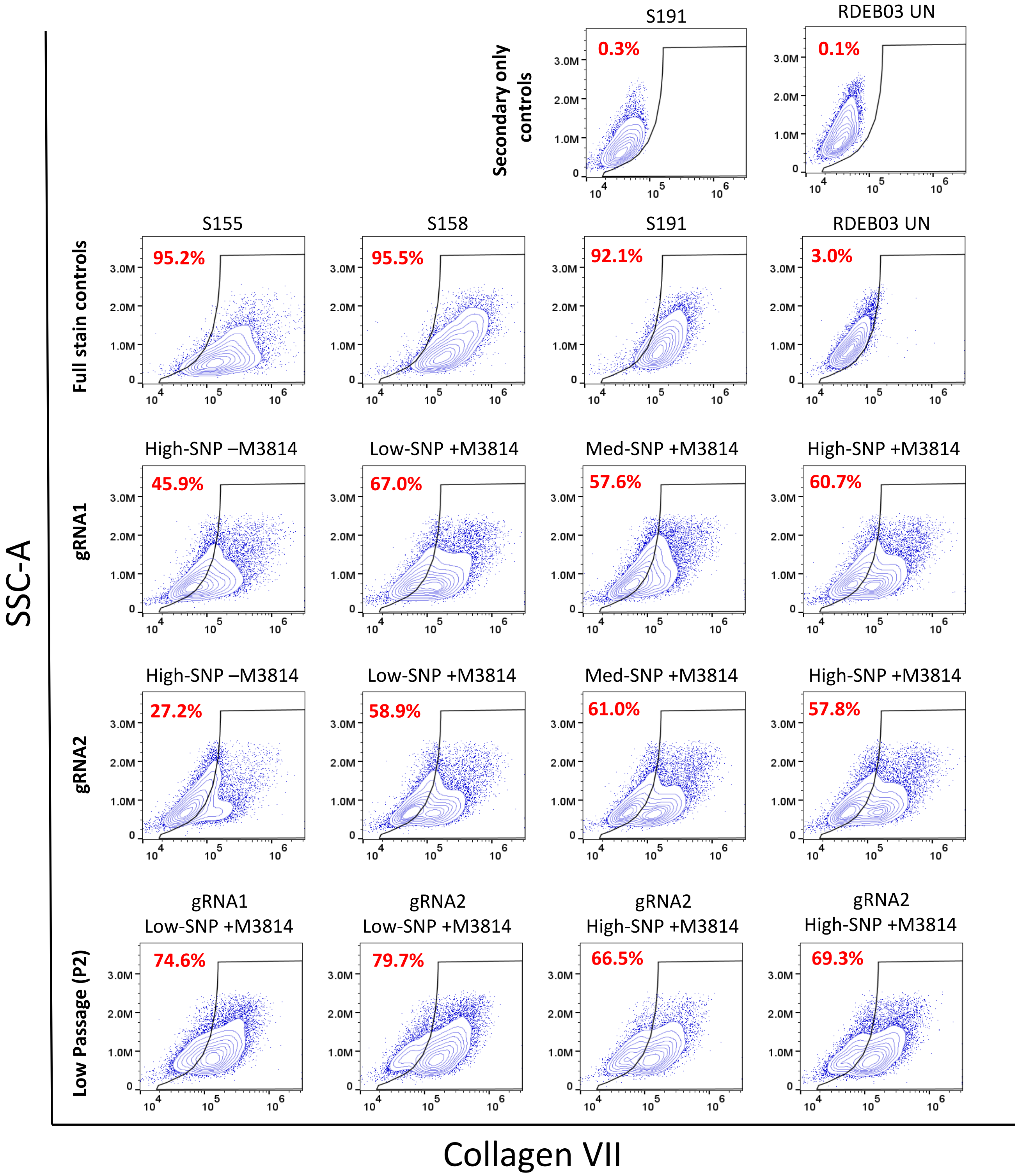

Figure S10: Quantification of C7 restoration in Cas9-nuclease edited RDEB03 keratinocytes. RDEB03 keratinocytes were edited with various gRNAs, HDR templates and M3814 exposure (as indicated) and were analyzed by flow cytometry to detect the restoration of C7 expression. Plots show C7 on the x-axis and side scatter (SSC-A) on the y-axis. The percentage of C7-positive cells is indicated in red text. S155, S158 and S191 are healthy donor keratinocytes which serve as positive controls. UN denotes unedited control.

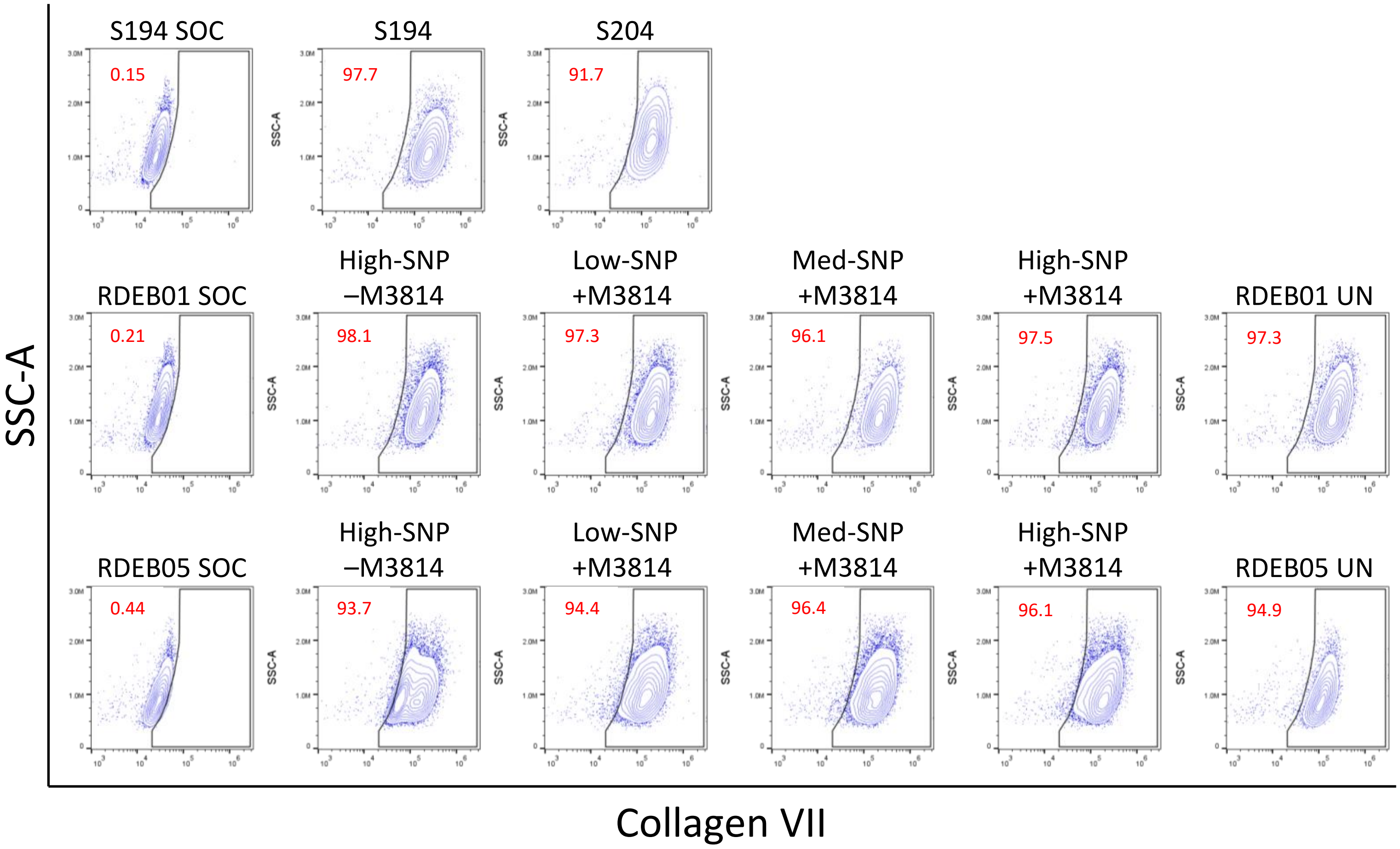

Figure S11: Quantification of C7 restoration in Cas9-nuclease edited RDEB01 and RDEB05 keratinocytes. RDEB01 and RDEB05 keratinocytes were edited with various HDR templates and M3814 exposure (as indicated) and were analyzed by flow cytometry to detect the restoration of C7 expression. Plots show C7 on the x-axis and side scatter (SSC-A) on the y-axis. The percentage of C7-positive cells is indicated in red text. S194 and S204 are healthy donor keratinocytes which serve as positive controls. SOC denotes secondary only controls and UN - unedited controls

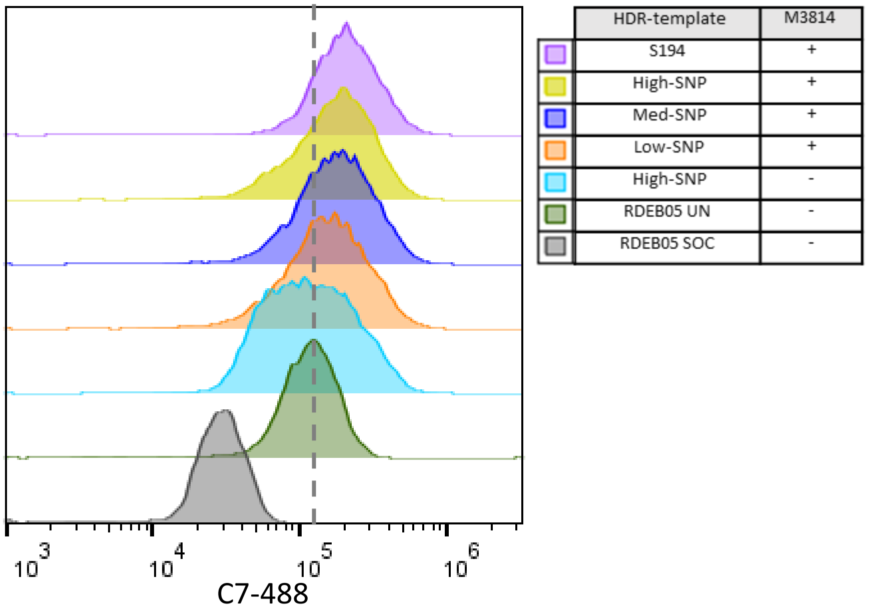

Figure S12: HDR editing of RDEB05 increases C7 fluorescence intensity. HDR editing of RDEB05 enhances C7 fluorescence intensity. RDEB05 keratinocytes edited with different HDR templates and M3814 exposure (as indicated) were analysed by flow cytometry. Histograms display C7-488 fluorescence intensity (x-axis). A grey dashed line marks the peak fluorescence intensity of RDEB05 unedited (UN) control. S194 serves as a positive control from healthy donor keratinocytes. SOC denotes secondary only control.

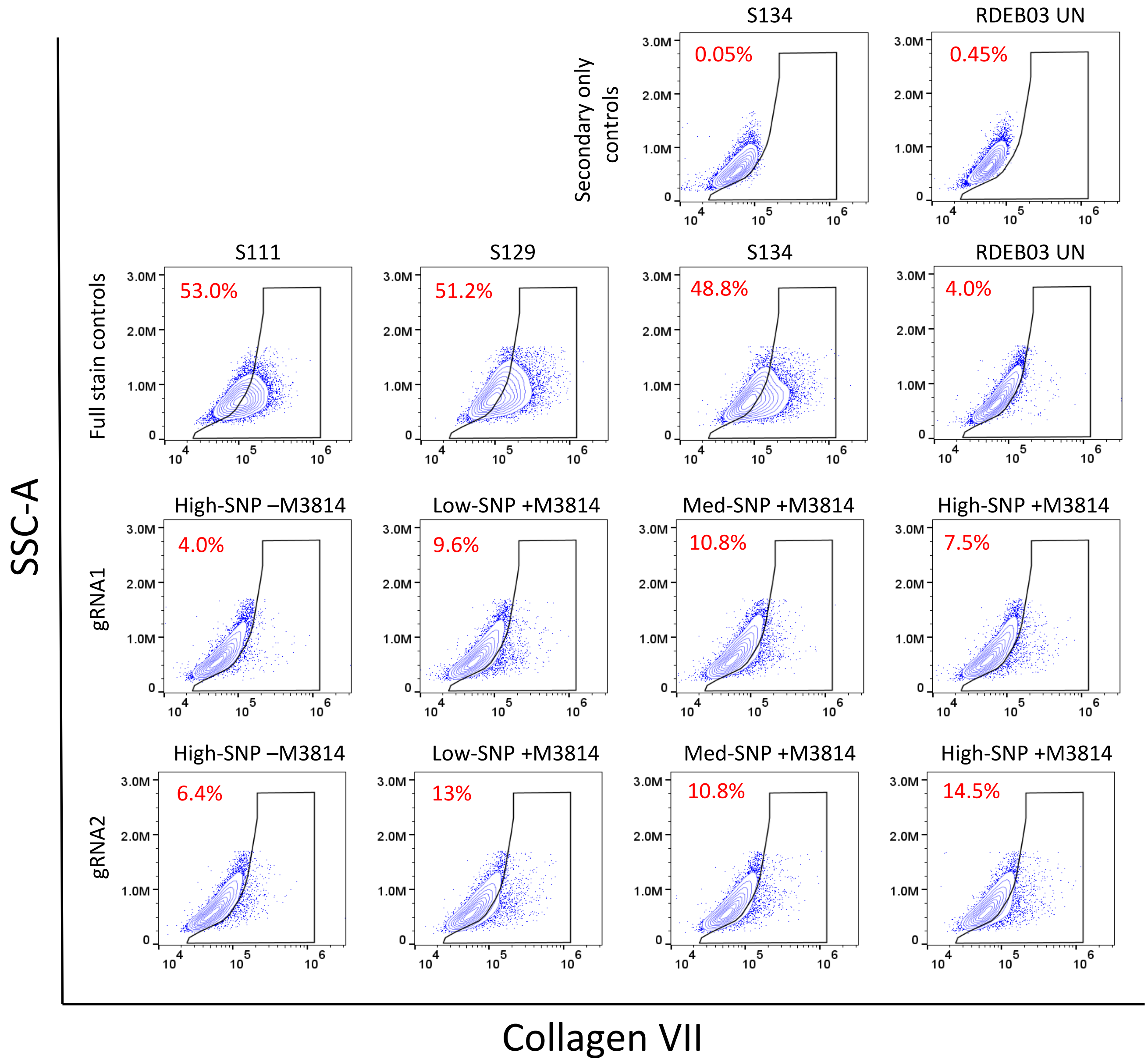

Figure S13: Quantification of C7 restoration in Cas9-nuclease edited RDEB03 fibroblasts. RDEB03 fibroblasts were edited with various gRNAs, HDR templates and M3814 exposure (as indicated) and were analyzed by flow cytometry to detect the restoration of C7 expression Plots show C7 on the x-axis and side scatter (SSC-A) on the y-axis. The percentage of C7-positive cells is indicated in red text. S111, S129 and S134 are healthy donor fibroblasts which serve as positive controls. UN denotes unedited control.

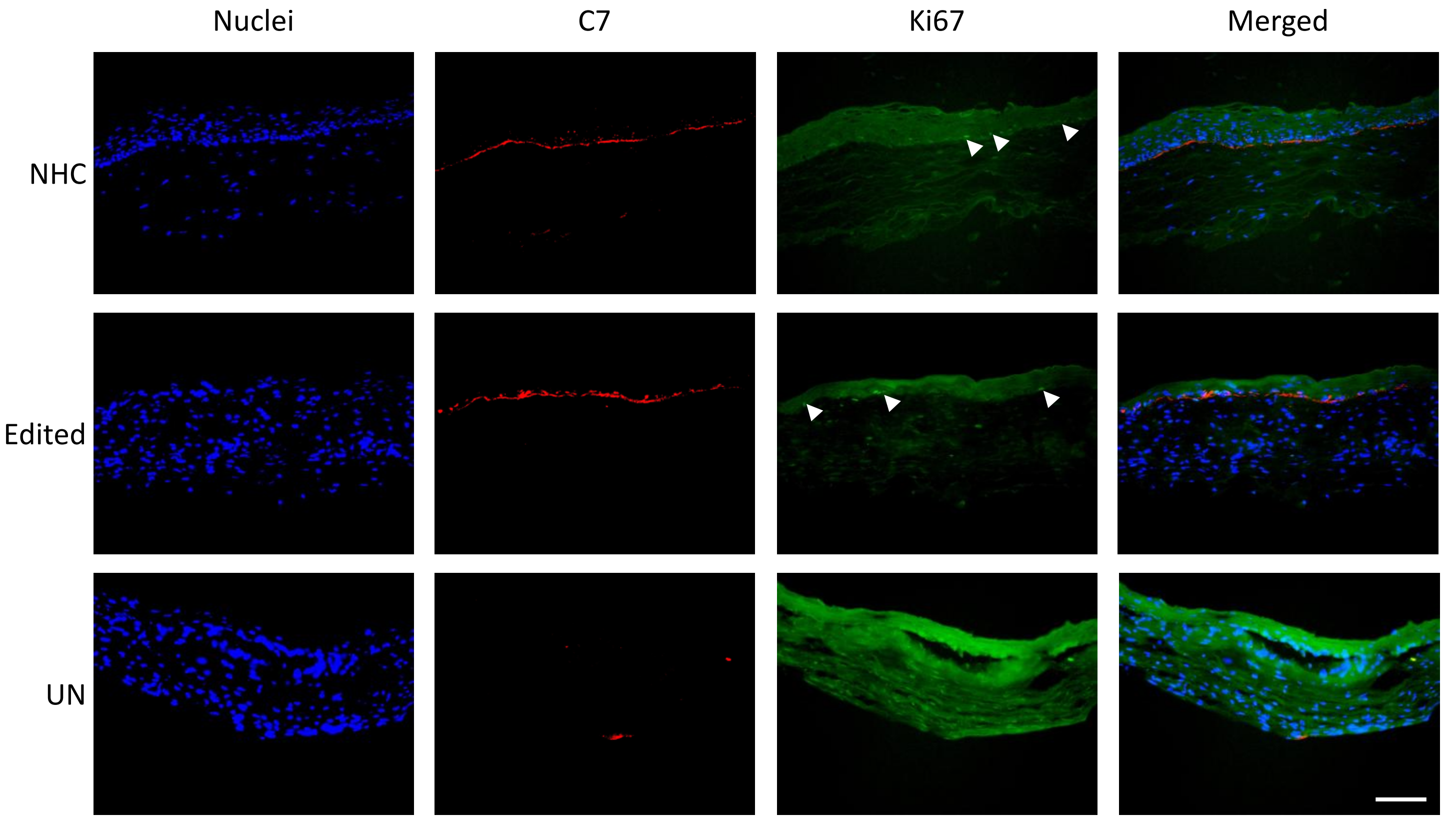

Figure S14: Immunohistochemistry of Ki67 positive basal epidermal keratinocytes in gene-edited 3D-bilayered skin equivalents. Low-passage (P2) RDEB03 cells edited with gRNA2 and the low-SNP template with M3814 were used to create bilayered skin equivalents. Representative immunohistochemistry (IHC) images from skin equivalents using normal human cells (NHC, top row), edited RDEB03 cells (middle row), and unedited RDEB03 cells (UN, bottom row) are shown. IHC analysis depicts C7 in red and the Ki67 proliferation marker in green. Nuclei are stained with DAPI (blue). Ki67-positive nuclei in NHK and edited RDEB03 sections are indicated by white arrows. High background fluorescence is observed in the Alexa Fluor 488 (Ki67) channel. Scale bar represents 100μM.

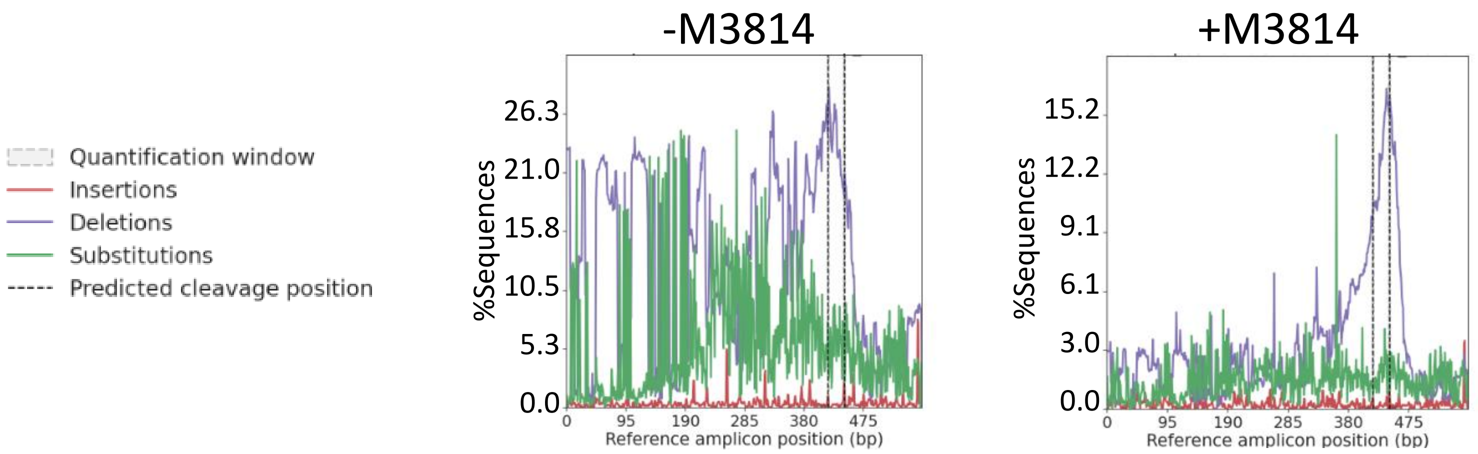

Figure S15: CRISPResso2 mutation distribution plot indicates that M3814 substantially decreases large mutations during paired-nickase editing at exon 117. Screen captures of indel distribution plots from paired-nickase edited RDEB03-Ks with and without M3814 as indicated. Plots show the location of the mutation on the x-axis and frequency of those mutations on the y-axis. Mutations are categorized by type, as displayed in the key.

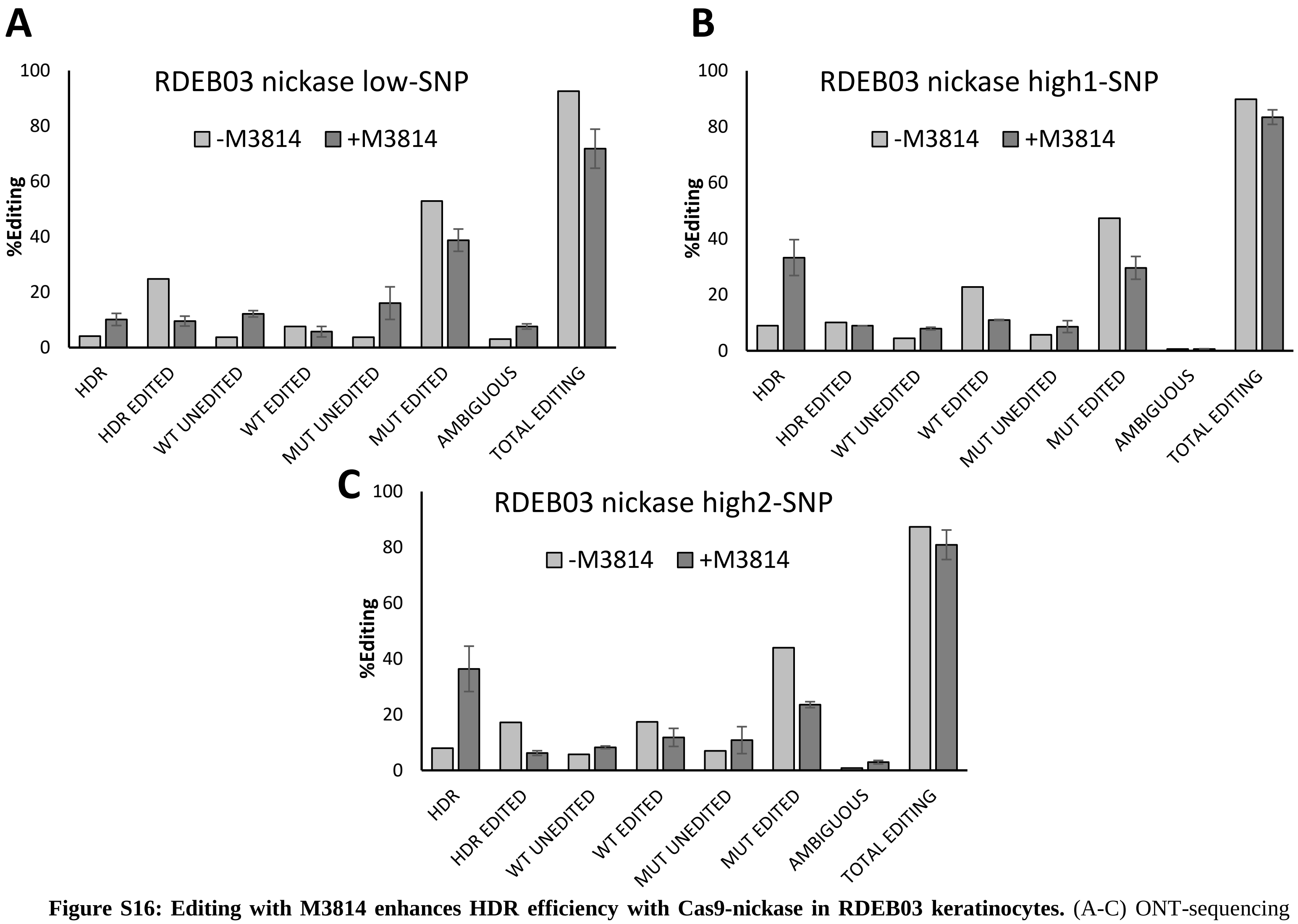

Figure S16: Editing with M3814 enhances HDR efficiency with Cas9-nickase in RDEB03 keratinocytes. (A-C) ONT-sequencing data are shown for RDEB03 keratinocytes edited with either the low-SNP (A), high1-SNP (B) or high2-SNP (C) HDR-templates with or without M3814. Data is shown as allele frequencies reported from CRISPResso2 analysis. Left to right: percentage HDR, HDR edited, wild-type (WT) unedited, WT edited, mutation (MUT) unedited, MUT edited and Ambiguous. MUT refers to the allele containing the targeted mutation (c.8698_8708del), while WT denotes the opposite allele. *n=2* for all conditions edited with M3814.

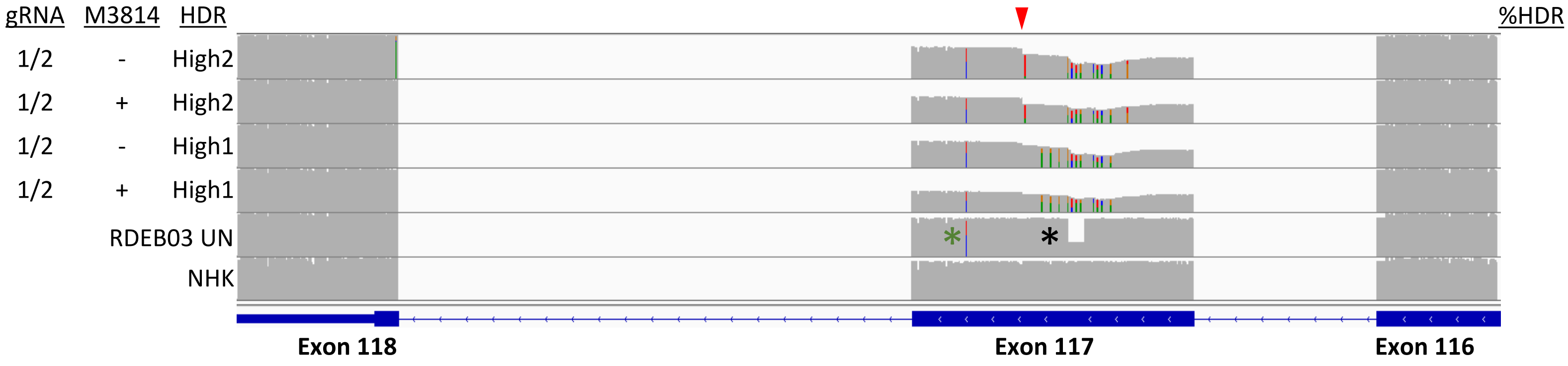

Figure S17: Analysis of *COL7A1* transcripts in paired-nickase edited RDEB03 keratinocytes reveals modified splicing of exon 117. ONT-seq was performed on PCR-amplified cDNA prepared from various edited RDEB03-K samples, and alignments displayed as coverage tracks in IGV. The sample conditions are listed on the left, where RDEB03 unedited (UN) and normal human keratinocytes (NHK) act as control sequences. The target 11bp deletion (c.8698_8708del) is indicated with a black asterisk, the green asterisk indicates the other, non-target mutation (c.8780G>A) on the opposite allele. The location of an aberrant splice site is marked by a red arrow. Exon 117 skipped transcripts are implied by reduced coverage of exon 117 compared to exon 116 and exon 118.

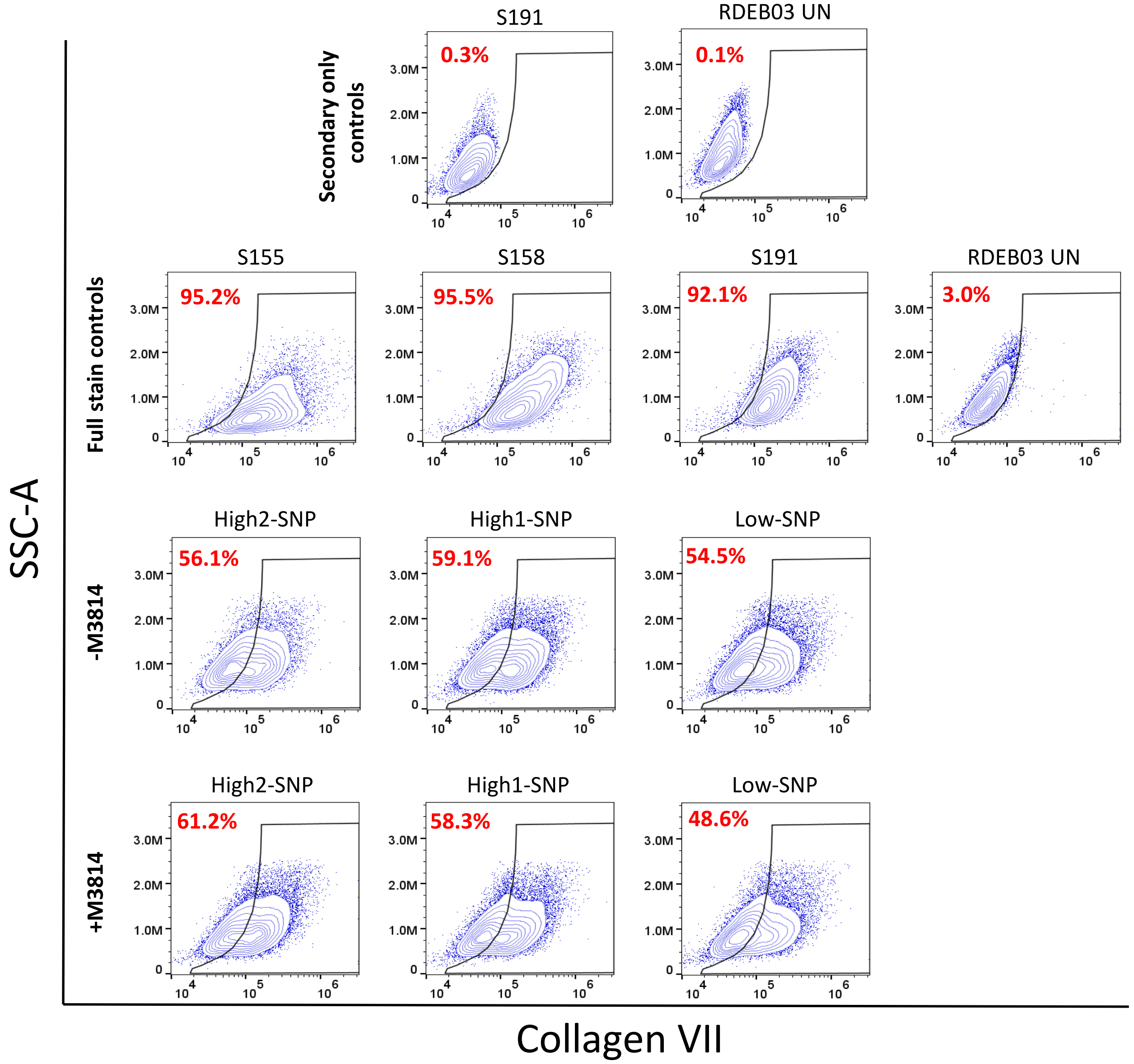

Figure S18: Quantification of C7 restoration in dual-Cas9 nickase edited RDEB03 keratinocytes. RDEB03 keratinocytes were edited with various HDR templates and M3814 exposure (as indicated) and were analyzed by flow cytometry to detect the restoration of C7 expression Plots show C7 on the x-axis and side scatter (SSC-A) on the y-axis. The percentage of C7-positive cells is indicated in red text. S155, S158 and S191 are healthy donor keratinocytes which serve as positive controls. UN denotes unedited control.

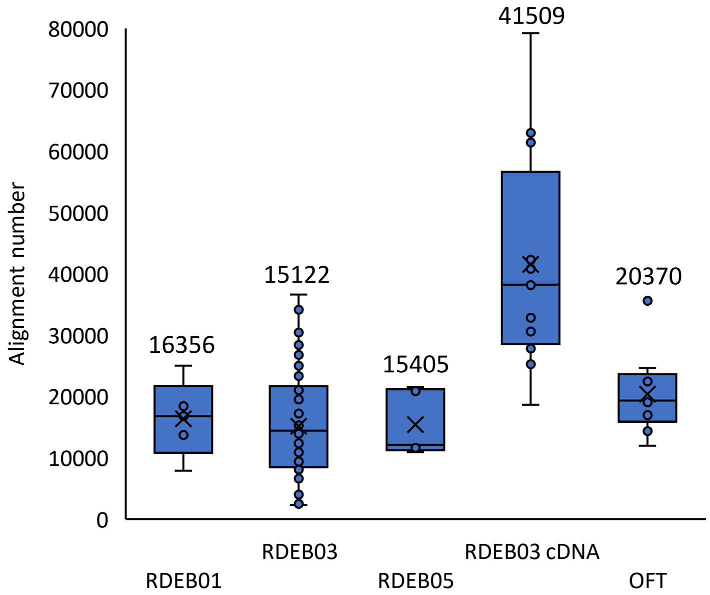

Figure S19: Average alignment number of ONT sequencing analysis. The alignment depths of all samples included in this analysis were compiled and categorised by PCR locus. From left to right: RDEB01-Ks (exon 21, *n=5*), RDEB03-Ks and Fs (exon 117, *n=56*), RDEB05-Ks (intron 26, *n=5*), RDEB03-Ks and Fs cDNA (exon 117, *n=12*), and off-target (OFT) sequences (variable loci, *n=12*). The mean alignment number for each locus is represented as a cross on the corresponding plot and is also listed above each plot.

Table S1: Primer pairs for amplification of on-target regions of *COL7A1* and off-target sites. (E, exon; I, intron; OFT, off-target).

Target	Name	Forward Primer (5'-3')	Reverse Primer (5'-3')
On-Target gDNA	*COL7A1* E117	CCCCCTGAGGATGATGAGTA	CACACAAGCCTCTAGCACCA
On-Target gDNA	*COL7A1* E21	GGAGGAAGTTAGGGACCATTG	GCGTCAGGGAGAAGATGTAAG
On-Target gDNA	*COL7A1* I26	TTGCAAAGGATCCGTGACAT	TGAGAGGACAAGTTGGGC
On-Target cDNA	*COL7A1* E117 cDNA	CTTCCATCAGGCTGGTCTGG	TCCTTTTCCAAGTGCCCTGA
On-Target cDNA	*COL7A1* I26 cDNA	TGCAGCCTCCTGTCTCTCTA	TCCTGGGACCCAACCAGTAT
Off-target	Cas9-g1 OFT1	GTGTACCGAAGGTCCCAAGG	AGCACAGTTCCGAGTCCAAG
Off-target	Cas9-g1 OFT2	AGAATTAGTGAGCGAGCGTCC	GCATTTAGTGGACCCGGAGG
Off-target	Cas9-g1 OFT3	ACTACCCGAAACAGAAGCCT	CAGGACCCACCCAGACAAAA
Off-target	Cas9-g1 OFT4	GAAATCCATTGCAGAGGGGC	GGTCCAAGTGTGACCTCCTC
Off-target	Cas9-g2 OFT1	CTGTTCTGCTCTGCCTCTCC	TCTGGTTGGCGGATGAGATG
Off-target	Cas9-g2 OFT2	GGATCAAGCCCTGCTCTACC	GGTCTAGTGTGACCACCTGC
Off-target	Cas9-g2 OFT3	GGCACGATTGCTCGTAGTCT	GGTCGTTTACGAGAGGCACA
Off-target	Cas9-g2 OFT4	GCGTATTTGGTTATCCGCCG	ACTGCCAGAAGTATGTCGCC
Off-target	Cas9nickase-g1 OFT1	GGCACGATTGCTCGTAGTCT	GGTCGTTTACGAGAGGCACA
Off-target	Cas9nickase-g1 OFT2	GCGTATTTGGTTATCCGCCG	ACTGCCAGAAGTATGTCGCC
Off-target	Cas9nickase-g1 OFT3	TGCCCCAGTATGGAAGAGGA	GCACAGGGGTCTGTGATACC
Off-target	Cas9nickase-g1 OFT4	TTTGGATCACCTTTGCGGTG	ACCCCACTTAGTCCTGTACGA
Off-target	Cas9nickase-g2 OFT1	TTTGGGGCTCACCTCTCAAC	CAGAGCAGCTATGCAGGTGT
Off-target	Cas9nickase-g2 OFT2	GCAACAGCTGCCTACTCCTT	CTGGCTATGCTGATCCCTGG
Off-target	Cas9nickase-g2 OFT3	ATCCAGGGCCACTTTGAGTC	GAAGGCCTCGGAAGGTCAAA
Off-target	Cas9nickase-g2 OFT4	GTAAGAGCCCTGCCATTGTG	AGACATAGTGCTACCTGAATGCTA

Table S2: gRNA sequences targeting *COL7A1*

Name	Sequence-PAM (5'-3')
RDEB03 gRNA1	ACAGGCCTGTCACAGCCCGA-TGG
RDEB03 gRNA2	ACCATAGACAAAAGGGTGAC-AGG
RDEB03 nickase-gRNA1	AGCCCGATGGTACCAGCGCA-GGG
RDEB03 nickase-gRNA2	TTGTCTATGGTGGCTGTGGA-GGG
RDEB01 gRNA1	GCCCGACACAGTACCGCGTG-AGG
RDEB05 gRNA1	TAATGTGGTGATGTTGGGAA-TGG

Table S3: HDR sequences for the correction of three *COL7A1* mutations

Name	Sequence (5'-3')
RDEB03 Low-SNP HDR	TGGATGAGGGCTCCTGCACTGCCTACACCCTGCGCTGGTACCATCGGGCTGTGACAGGCAGCACAGAGGCTTGTCACCCTTTTGTCTATGGTGGCTGTGGAGGGAATGCCA
RDEB03 Med-SNP HDR	TGGATGAGGGCTCCTGCACTGCCTACACCCTGCGCTGGTACCATCGGGCAGTGACAGGCAGCACTGAGGCTTGTCACCCTTTTGTCTATGGTGGCTGTGGAGGGAATGCCA
RDEB03 High-SNP HDR	TGGATGAGGGCTCCTGCACTGCCTACACCCTGCGCTGGTATCATCGTGCAGTTACAGGCAGTACTGAAGCTTGTCACCCTTTTGTCTATGGTGGCTGTGGAGGGAATGCCA
RDEB03 nickase Low-SNP HDR	TGGATGAGGGCTCCTGCACTGCCTACACCCTGCGCTGGTATCATCGGGCTGTGACAGGCAGCACAGAGGCCTGTCACCCTTTTGTCTACGGTGGCTGTGGAGGGAATGCCAACCGTTTTGGGACCCGTG
RDEB03 nickase High1-SNP HDR	TGGATGAGGGCTCCTGCACTGCCTACACCCTGCGCTGGTATCATCGTGCAGTTACAGGCAGTACTGAAGCTTGTCATCCTTTCGTCTACGGTGGCTGTGGAGGGAATGCCAACCGTTTTGGGACCCGTG
RDEB03 nickase High2-SNP HDR	CTGCCACTGGATGAGGGCTCCTGCACTGCCTACACACTGCGCTGGTATCATCGTGCAGTTACAGGCAGTACTGAAGCTTGTCACCCTTTTGTCTATGGTGGCTGTGGTGGGAATGCCAACCGTTTTGGGACCCGTGAGGCCTG
RDEB01 Low-SNP HDR	CAGGAACAGTCCCGGGTCCTGGGGCCCGAGCTCAGCAGCTATCACCTGGACGGGCTGGAGCCAGCGACACAGTACCGCGTGAGACTGAGTGTCCTAGGGCCAGCTGGAGAAGGGCCC
RDEB01 Med-SNP HDR	CAGGAACAGTCCCGGGTCCTGGGGCCCGAGCTCAGCAGCTATCACCTGGACGGGCTGGAGCCAGCGACCCAGTATCGCGTGAGACTGAGTGTCCTAGGGCCAGCTGGAGAAGGGCCC
RDEB01 High-SNP HDR	CAGGAACAGTCCCGGGTCCTGGGGCCCGAGCTTAGCAGCTATCACCTGGACGGGCTGGAGCCTGCAACCCACTATCGGGTGAGACTGAGTGTCCTAGGGCCAGCTGGAGAAGGGCCC
RDEB05 Low-SNP HDR	GGCTGATGGGGAAGGGGTCTGGGAATAGGGGTGGCCCTGAAAAGGCTATCATGCAGCCACTGGACCCCACCTTAGGGCTTAATGTGGTGATGCTGGGCATGGCTGGAGCGGACCCAGAGCAGCTGCGTCGCTTGGCGCCGGGTAT
RDEB05 Med-SNP HDR	GGCTGATGGGGAAGGGGTCTGGGAATAGGGGTGGCCCTGAAAAGGCTATCATGCAGCTACTGGACCCCACCTTAGGGCTGAATGTGGTGATGCTGGGCATGGCTGGAGCGGACCCAGAGCAGCTGCGTCGCTTGGCGCCGGGTAT
RDEB05 High-SNP HDR	GGCTGATGGGGAAGGGGTCTGGGAATAGGGGTGGCTCTGAAAAGGCTATCATGCAGCTACTGGACCCCATCTTAGGCCTGAACGTCGTGATGCTGGGCATGGCTGGAGCCGACCCAGAGCAGCTGCGTCGCTTGGCGCCGGGTAT
